# Photoacoustic device fingerprints induce bias in deep learning models

**DOI:** 10.1038/s41598-026-53468-6

**Published:** 2026-06-13

**Authors:** Christoph J. Bender, Marcel Knopp, Niklas Holzwarth, Tom Rix, Jan-Hinrich Nölke, Kris K. Dreher, Yi Li, Julius Kempf, Milenko Caranovic, Fabian Schneider, Melanie Schellenberg, Leonie Boland, Briain Haney, Ferdinand Knieling, Ulrich Rother, Alexander Seitel, Lena Maier-Hein

**Affiliations:** 1 Division of Intelligent Medical Systems (IMSY), German Cancer Research Center (DKFZ) Heidelberg, Heidelberg, Germany; 2 Medical Faculty, Heidelberg University, Heidelberg, Germany; 3 Faculty of Mathematics and Computer Science, Heidelberg University, Heidelberg, Germany; 4 Faculty of Physics and Astronomy, Heidelberg University, Heidelberg, Germany; 5 Division of Medical Image Computing (MIC), German Cancer Research Center (DKFZ) Heidelberg, Heidelberg, Germany; 6 Helmholtz Imaging, German Cancer Research Center (DKFZ), Heidelberg, Germany; 7 Department of Vascular Surgery, University Hospital Erlangen, Friedrich-Alexander-Universität Erlangen-Nürnberg (FAU), Erlangen, Germany; 8 Institute of Biological and Medical Imaging, Helmholtz Zentrum München, Neuherberg, Germany; 9 Chair of Biological Imaging, Central Institute for Translational Cancer Research (TranslaTUM), School of Medicine and Health, Technical University of Munich, Munich, Germany; 10 Department of Medical Physics, Memorial Sloan Kettering Cancer Center, New York, USA; 11 Department of Pediatrics and Adolescent Medicine, University Hospital Erlangen, Friedrich-Alexander-Universität Erlangen-Nürnberg (FAU), Erlangen, Germany; 12 National Center for Tumor Diseases (NCT), NCT Heidelberg, a partnership between DKFZ and University Hospital Heidelberg, Heidelberg, Germany; 13 Surgical AI Research Group, Heidelberg University Hospital, Surgical Clinic, Heidelberg, Germany; 14 HIDSS4Health – Helmholtz Information and Data Science School for Health, Karlsruhe/Heidelberg, Germany; 15 Mohamed Bin Zayed University of Artificial Intelligence (MBZUAI), Abu Dhabi, UAE

**Keywords:** Deep learning, Photoacoustic, Shortcut learning, Bias, Hardware confounder, Biomarkers, Computational biology and bioinformatics, Engineering, Health care, Medical research

## Abstract

Deep learning (DL) models developed for established medical imaging modalities have shown increasing performance and reliability as a result of scaling efforts. In contrast, model development for emerging modalities such as photoacoustic imaging (PAI) remains challenged by data sparsity, which limits model generalizability and raises the susceptibility to bias. While recent studies in PAI have started to investigate subject-related confounders, the impact of hardware-related confounders remains unexplored, posing a critical risk for failure in multicentric deployment scenarios. We are the first to provide a multicentric analysis of hardware-induced bias in PAI. We analyzed device-specific characteristics in images from four device instances and two peripheral artery disease studies, and trained DL models to classify device origin and disease under varying levels of device–health correlations in the data. We showed that 1) multiple instances of the same PAI device type embed identifiable fingerprints in the images, 2) that DL models can leverage these fingerprints to reach $100\,\%$ accuracy in device detection and critically, 3) when a correlation between device instance and health status is present, models trained for disease diagnosis exploit these device-specific signatures as shortcuts, thereby producing biased and clinically misleading predictions. This research highlights the risk of overestimating algorithm performance when such confounding is overlooked, emphasizing the importance of bias evaluation and explainable artificial intelligence methods to identify potential shortcuts, finally enabling multicentric PAI studies.

## Introduction

Deep learning (DL) is increasingly used in medical image analysis to support diagnosis, disease characterization, and clinical decision support. Despite this growing adoption, translating DL models into reliable clinical tools remains a major challenge. One of the principal barriers is the tendency of models to learn from biases inherent in the data, which can lead to unreliable, unfair, and clinically misleading predictions^1–3^. Numerous studies have shown that even subtle biases stemming from demographic, institutional, or technical factors can significantly impair model generalizability and validity^4–8^. Understanding and mitigating such biases is therefore crucial for the safe and fair deployment of medical artificial intelligence (AI) systems.

In established imaging modalities such as magnetic resonance imaging (MRI) and chest radiography, it is well documented that variations in scanner hardware or acquisition protocols can act as confounders^7,9,10^. These variations often induce “shortcut learning”^1^, whereby spurious correlations in the training data lead neural networks to exploit non-task-relevant cues, such as scanner-specific artifacts or acquisition settings, instead of genuinely task-relevant features like disease-related image patterns. As a result, models may achieve putative high performance on internal datasets that preserve the same confounding structure but fail when applied on data where this spurious correlation is absent. While such hardware-induced biases are increasingly recognized in mature imaging modalities, their impact in emerging technologies such as photoacoustic imaging (PAI) remains largely unexplored.

PAI is a hybrid optical and acoustic modality that combines pulsed optical excitation at multiple wavelengths with ultrasonic detection to visualize the spatial distribution of light absorption in tissue. By exploiting the photoacoustic effect and multispectral excitation, PAI enables noninvasive mapping of functional biomarkers, such as hemoglobin concentration and blood oxygenation, based on their distinct optical absorption spectra. Its ability to provide both structural and functional information positions PAI as a promising tool for diverse clinical applications, including cancer detection, vascular imaging, monitoring of blood oxygen saturation, and the monitoring of tissue perfusion^11–15^. However, as a relatively new modality with inherently sparse datasets, models trained on PAI data might be particularly susceptible to biases arising from nonstandardized hardware and acquisition protocols.

Addressing hardware confounders is critical for the clinical translation of PAI, especially as forthcoming large-scale, multi-center studies will rely on data from multiple device instances. Such datasets inherently risk introducing subtle, device-specific “fingerprints”, i.e., characteristic noise patterns or spectral signatures that uniquely identify each system. In PAI, where hardware components and acquisition settings vary and datasets remain relatively small, these fingerprints can easily correlate with disease labels. As a result, DL models risk inheriting device-specific biases that limit their generalizability.

Although previous studies have begun to investigate physiological and demographic confounders in PAI^16,17^, we are not aware of any study that has systematically investigated hardware-induced bias.

**Fig. 1:**
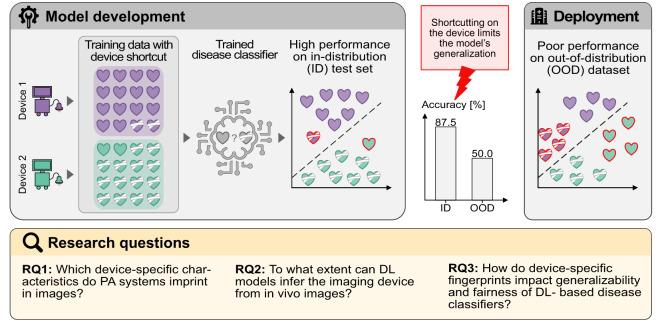
Device fingerprints can bias photoacoustic disease classifiers. Top: Deep learning (DL) models trained on photoacoustic (PA) images may rely on device-specific fingerprints rather than disease-related features. When training data contain a spurious correlation between device instance and health status in model development, the resulting classifier shows seemingly high accuracy on in-distribution (ID) test sets but fails to generalize to out-of-distribution (OOD) data at the deployment stage. Bottom: research questions (RQs) addressed in the paper.

To close this gap, we systematically conduct the following investigations, which are important for advancing reliable, bias-aware DL in PAI and for paving the way toward its clinical translation (see research questions defined in Fig. 1):

1. Characterizing the extent to which device instances embed distinct hardware-specific fingerprints.
2. Determining the detectability of these fingerprints in vivo by DL models.
3. Investigating how these device-dependent signals induce shortcut learning in disease-classification models when device–health correlations are present.

Together, these investigations establish a systematic foundation for understanding how hardware variability affects DL-based PAI analysis, addressing the practically important and so far unresolved question of how hardware-related confounders in data pooled across nominally identical device instances may hinder transferable and fair downstream prediction models in future multicenter studies, and how such effects should be considered in study design and analysis.

## Results

Our findings are presented in the following. Details on experimental design, data acquisition, and cohort composition can be found in Materials and methods.

### Photoacoustic systems embed device-specific fingerprints

**Fig. 2:**
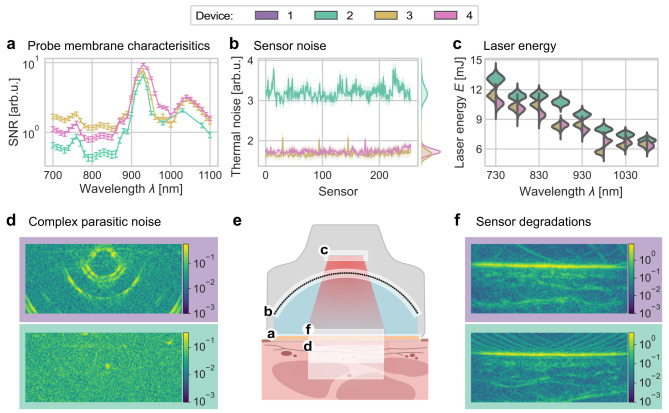
Photoacoustic imaging devices feature distinct device fingerprints despite identical system type. Although all four device instances investigated were of the same model (MSOT Acuity Echo), several device-specific characteristics were observed. In aqua experiments with devices 2–4 revealed differences in (**a**) probe membrane signal-to-noise ratios (SNR), with error bars indicating three times the standard deviation; (**b**) thermal sensor noise levels, shown with shaded error bands representing one standard deviation; and (**c**) laser energy distributions across wavelengths. In vivo imaging demonstrated systematic artifacts, including (**d**) complex parasitic noise producing ring-shaped patterns in device 1 but not in device 2, and (**f**) sensor degradations resulting in streaking artifacts arising from broken sensors in device 1 and from sensors with shifted temporal responses in device 2. **e** Schematic of the MSOT imaging geometry, showing the laser illumination (red), transducer array (dotted), coupling medium (light blue), membrane (orange), and underlying tissue layers. White boxes indicate where device-specific signal artifacts originate (**a-c**) or how they manifest within the standard photoacoustic field of view (**d & f**).

Across the four device instances examined, we identified distinct hardware-specific signatures specifically related to the image formation process that collectively form noticeable device fingerprints. The detected fingerprints comprised: **a** variations in the signal-to-noise ratio (SNR) of the probe membrane, **b** differences in thermal sensor noise levels, **c** device-specific laser energy profiles, **d** existence or absence of complex parasitic noise patterns, and **f** sensor degradation effects (Fig. 2). In addition to differences in membrane SNR (Fig. 2a), further membrane-related discrepancies, such as systematic depth offsets arising from different amounts of acoustic couplant, are described in the Supplementary (Fig. S1 and S2). Together, these findings indicate that PAI systems carry measurable device-intrinsic features that persist across measurement contexts and can influence downstream analyses.

### Device classification models robustly identify the device origin from in vivo images despite image corrections

**Fig. 3:**
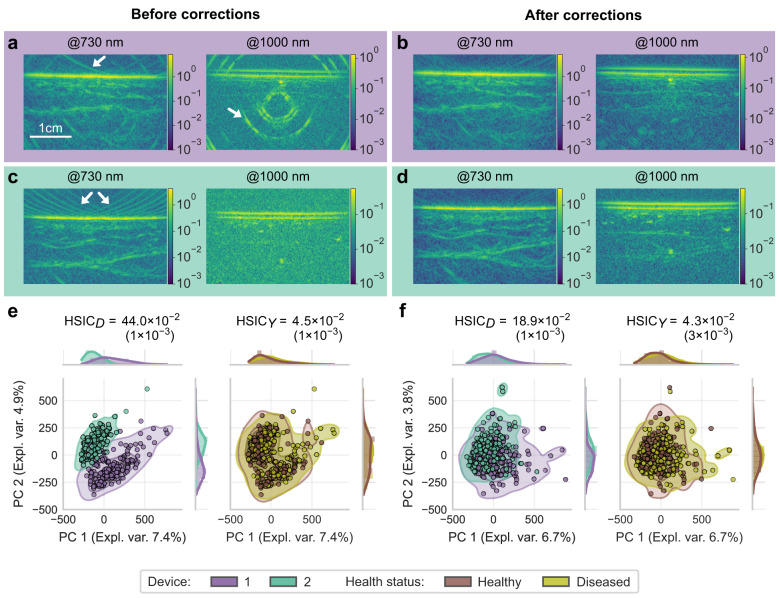
Image-based corrections reduce systematic device-specific fingerprints in in vivo photoacoustic images. Device-specific artifacts were observed in the in vivo dataset, including (**a**) ring-shaped parasitic noise and streaking artifacts due to broken sensors in device 1, and (**c**) multiple streaking patterns due to early response sensors in device 2. (**b, d**) A multi-step correction pipeline (detailed in Signal correction methods) reduced these visible artifacts in both devices. (**e, f**) Projections of the in vivo images onto the first principal components (PCs) are shown color-coded by device (left) and health status (right). In the PCA embedding, the normalized Hilbert-Schmidt Independence Criterion ($\textrm{nHSIC}$) was computed to quantify dependence on device instance ($\textrm{nHSIC}_D$) and health status ($\textrm{nHSIC}_Y$). The PC projections revealed clearly separable device-specific clusters before correction (**e**), which converge substantially after correction (**f**), demonstrating a notable reduction of device-specific fingerprints, whereas health status did not show notable clustering either before or after correction. This qualitative change is quantitatively supported by a decrease in $\textrm{nHSIC}_D$ from 0.440 in uncorrected images to 0.189 after correction, while $\textrm{nHSIC}_Y$ remained comparatively low and nearly unchanged (0.045 vs. 0.043). $\textrm{nHSIC}$ was calculated on PCs explaining $80\,\%$ of the variance. Numbers in brackets denote the p-value computed via permutation tests with 1000 repetitions.

While device-specific fingerprints were visually apparent for in aqua and non-corrected in vivo image data (Fig. 2), their relevance for machine learning models warrants investigation. Principal component analysis (PCA) of the in vivo dataset revealed distinct, separable clusters for each device (Fig. 3e), confirming the prominent device-dependent artifacts (Fig. 3a/c). In device 1, pronounced streaking artifacts stemmed from broken sensors ($n_b \in \{31, 48, 49, 158\}$), particularly the central sensor $n_b=158$, alongside extensive parasitic ring noise (white arrows in Fig. 3 **a**). Device 2, by contrast, exhibited streaking artifacts due to early response sensors, where every eighth sensor produced signals shifted $\delta t = 0.5 - 2.5$ time steps too early (white arrows in Fig. 3c).

Following correction, comprising (i) early response sensor correction, (ii) singular value decomposition–based reduction of complex parasitic noise, (iii) temporal averaging, (iv) broken sensor interpolation, and (v) depth alignment (see Signal correction methods), the device-specific PCA clusters converged markedly (Fig. 3f), reflected in a decrease in normalized Hilbert-Schmidt Independence Criterion (nHSIC)^18–20^ with respect to device instance, denoted as $\textrm{nHSIC}_D$, from 0.440 to 0.189. nHSIC was calculated on principal components explaining $80\,\%$ of the variance using Eq. (2). In addition, the $\textrm{nHSIC}_Y$ for the health status given in the in vivo dataset remained comparatively low and essentially unchanged after correction (0.045 vs. 0.043). This observation suggests, first, that the corrections do not remove substantial disease-related signals and, second, that even after correction, device system identity is still more strongly encoded in the data than clinically relevant pathology.

**Fig. 4:**
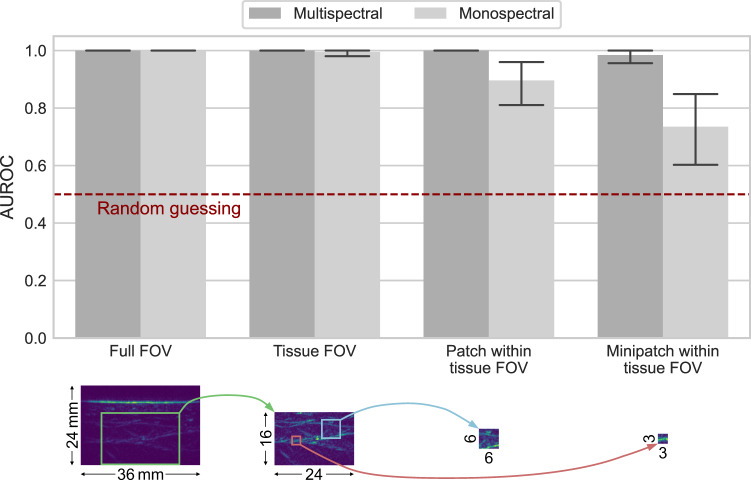
Device classification models reliably identify device origin. Deep learning model ensembles were trained to classify device origin from corrected in vivo photoacoustic images under varying spatial fields of view (FOV) and spectral configurations (multispectral and monospectral at $\lambda = 800\, \textrm{nm}$). Bars show the mean area under the receiver operating characteristic curve (AUROC) with whiskers indicating the $95\,\%$ confidence intervals (CIs). The bottom row illustrates, for one exemplary subject at $\lambda = 800\, \textrm{nm}$, the corresponding FOV and patch locations; the labels 36 $\times$ 24, 24 $\times$ 16, 6 $\times$ 6, and 3 $\times$ 3 indicate the physical patch dimensions in millimeters along the x- and y-axes of the respective image crops.

Despite the substantial reduction of visible artifacts, the remaining device-specific fingerprints were still distinctive enough for a device classification model to reliably identify device origin, with an area under the receiver operating characteristic curve (AUROC) of 1.0 for both multispectral and monospectral images on the full field of view (FOV) (Fig. 4). This was the case even when excluding regions known to contain device-related variability (e.g., pixels above the skin affected by streaking artifacts, or pixels at higher wavelengths with pronounced system-specific differences), and even when training on increasingly small image patches, where AUROC values were as high as 1.0 down to tissue FOV and patch level and remained at 0.98 ($95\,\%$ $\mathrm{CI}: 0.96-1.0$) for multispectral minipatches and 0.74 ($95\,\%$ $\mathrm{CI}: 0.60-0.85$) for monospectral minipatches (Fig. 4). The FOV included the supra-skin region, whereas the restricted tissue FOV contained only tissue pixels. Additional random crops further minimized the input area to about $4\,\%$ and $1\,\%$ of the original spatial information, respectively.

For multispectral images, spatial cropping had minimal effect on performance, with AUROC values consistently $\ge$ 0.98. For monospectral images at $800\,\textrm{nm}$, classification accuracy decreased with smaller patches but remained far above random guessing, achieving AUROC values of 0.89 ($95\,\%$ $\mathrm{CI}: 0.81-0.96$) for $6\,\textrm{mm}\times6\,\textrm{mm}$ patches and 0.74 ($95\,\%$ $\mathrm{CI}: 0.60-0.85$) for $3\, \textrm{mm} \times 3\, \textrm{mm}$ patches.

Having established that device detection models can recover device identity from corrected in vivo images, we next examined whether disease classification models exploit these same device fingerprints as shortcuts when predicting health status.

### Disease classification models can exhibit shortcut learning due to overreliance on device fingerprints rather than disease-related features

**Fig. 5:**
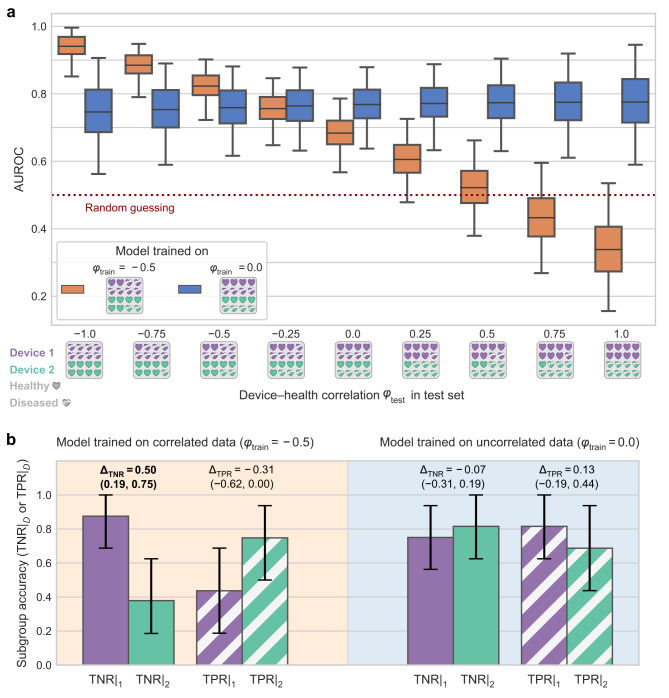
Spurious device–health correlations mislead disease classifiers. Two deep learning (DL) model ensembles were trained on the in vivo full field of view (FOV) dataset to predict health status (i.e. whether or not a patient has peripheral artery disease): one with a negative device–health correlation ($\varphi _{\text {train}} = -0.5$, orange) and one with uncorrelated data ($\varphi _{\text {train}} = 0$, blue). (**a**) Area under the receiver operating characteristic (AUROC) curve values were calculated depending on varying degrees of device shortcut $\varphi _{\text {test}}$ (defined in Eq. (4)). The correlated model performed well when the test sets preserved or amplified the shortcut ($\varphi _{\text {test}} \le -0.5$) but declined steadily as the correlation weakened or reversed. In contrast, the uncorrelated model maintained stable performance across all test sets. (**b**) True negative rate (TNR) and true positive rate (TPR) across the two devices were calculated for both models. These subgroup accuracies revealed strong disparities in specificity ($\Delta _{\textrm{TNR}}$) and sensitivity ($\Delta _{\textrm{TPR}}$) for the correlated model, indicating a bias toward predicting positives on device 1 and negatives on device 2. The uncorrelated model exhibited no significant subgroup differences, suggesting fair performance across devices. Whiskers and numbers in brackets indicate the $95\,\%$ confidence intervals for the subgroup accuracies and fairness metrics, respectively. Bold disparity values indicate that the fair reference value ($\Delta =0$) lies outside the corresponding confidence interval.

Performance of the disease classification models strongly depended on the presence of device shortcuts in the test data (Fig. 5a). Here, the phi coefficient $\varphi$^21,22^ describes the strength of the device–health correlation, with $\varphi =0$ meaning no correlation and $|\varphi | \rightarrow 1$ strong correlation. The disease classifier trained on the correlated dataset ($\varphi =-0.5$) performed well only when the test data preserved or amplified the shortcut. However, its AUROC dropped sharply once the test set’s device–health association diverged from that of the training set. In contrast, the uncorrelated model maintained stable performance across all test sets. Consistent with this observation, the performance skewness increased with stronger training correlations as demonstrated by the AUROC scores and balanced accuracy (BA) values for models trained on $\varphi _\text {train}\in [-1,1]$ and evaluated for different $\varphi _\text {test}\in [-1,1]$, which are shown in the Supplementary (Fig. S3 and S4). Peripheral artery disease (PAD) classifier performance across the four device–health subgroups showed disparities in sensitivity or true positive rate (TPR) and specificity or true negative rate (TNR). Namely, the biased model reflected a systematic bias toward predicting positives on one device and negatives on another. The model trained on uncorrelated data showed no major subgroup disparities, demonstrating fair behavior across devices (Fig. 5b).

**Fig. 6:**
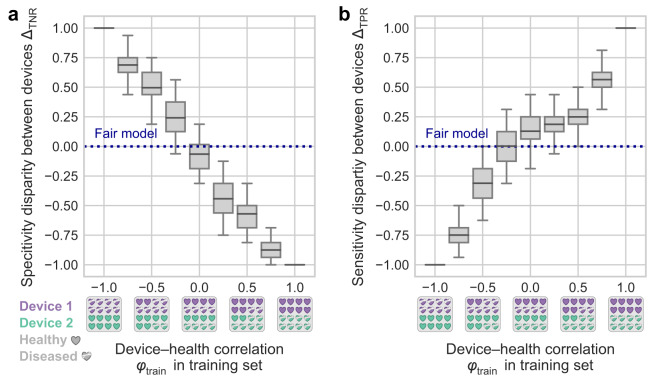
Stronger device shortcuts in the training data produce increasingly biased disease classifiers. Model ensembles were trained to predict the health status for different levels of health status-device correlation in the training data. (**a**) Specificity disparity ($\Delta _{\textrm{TNR}}$) and (**b**) sensitivity disparity ($\Delta _{\textrm{TPR}}$) quantify fairness across device subgroups. A value of $\Delta =0$ (blue dotted line) indicates perfect fairness, meaning equal performance for both devices. Model ensembles trained without device shortcuts showed no notable deviation from $\Delta =0$. As $|\varphi _{\text {train}}|$ increased, the absolute disparity in both metrics grew, reaching a disparity $|\Delta |=1$ under maximal correlation, indicating predictions based solely on device origin. Whiskers represent $95\,\%$ confidence intervals.

The extent of these disparities ($\Delta _{\textrm{TNR}}$, $\Delta _{\textrm{TPR}}$) depended on the strength of the device–health correlation in the training data. As $|\varphi _{\text {train}}|$ increased, the disease classifiers became increasingly biased (Fig. 6). Under maximal spurious correlation, models reached perfect disparity ($|\Delta |=1$) in both sensitivity and specificity, indicating complete reliance on device-specific features rather than clinically relevant patterns.

**Fig. 7:**
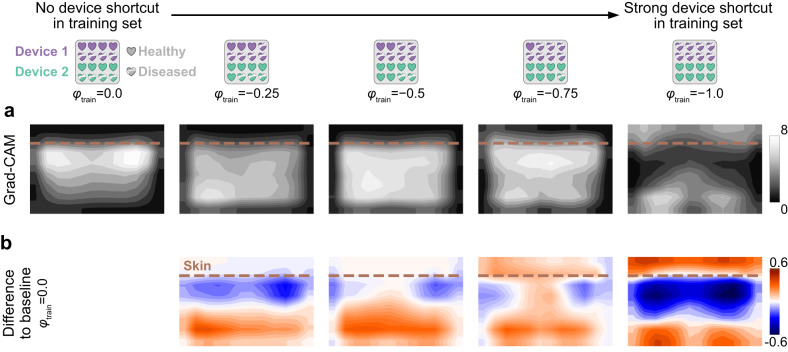
Device shortcuts shift disease classifiers’ focus to irrelevant features. (**a**) Gradient-weighted Class Activation Maps (Grad-CAMs) for models trained with different levels of spurious correlation, from a baseline ($\varphi _{\text {train}}=0$) to a strong shortcut ($\varphi _{\text {train}}=-1.0$) in steps of 0.25, averaged over all samples of a balanced test set. The larger (white) the Grad-Cam values are, the more relevant the region is for the model’s decision. (**b**) Paired differences between each shortcut-trained model and the baseline model ($\varphi _{\text {train}}=0$) averaged over all test set samples illustrate consistent shifts in model attention. All models trained with device shortcuts increasingly focused on pixels deep within the tissue, a region known to be prone to noise. With increasing spurious correlations, the models focus even more on clearly disease-irrelevant features, such as pixels above the skin. At $\varphi _{\text {train}}=1.0$, an arc-shaped pattern characteristic of complex parasitic noise became dominant in the model’s attention. The dashed green line indicates the skin level in the photoacoustic image.

Gradient-based Class Activation Maps (Grad-CAMs)^23^, computed with the Grad-CAM variant High-Resolution Class Activation Mapping (HiResCAM)^24,25^, revealed that with no device shortcut in the training data ($\varphi _{\text {train}}=0$), models primarily focused on central image regions where physiological differences in the tissue are expected. However, with increasing shortcut strength, attention progressively shifted toward image areas known to encode device fingerprints, for example, regions above the skin and peripheral areas where system-specific noise is most visible (Fig. 7). At $\varphi _{\text {train}}=-1.0$, models consistently focused on a characteristic arc-shaped pattern corresponding to complex parasitic noise. Generally these regions correspond to the device-specific artifact patterns shown in Fig. 3.

To further investigate whether learned representations encode device- or disease-related information, we computed the nHSIC with respect to device identity ($\textrm{nHSIC}_D$) and health status ($\textrm{nHSIC}_Y$) across different network layers, alongside visualizing PCA embeddings. These analyses show that early-layer representations exhibited significant device dependence across all training settings ($\varphi _\text {train}$), clearly exceeding health status dependence in the corresponding layers (see Supplementary Figs. S5 and S7). Across network depth, the evolution of the learned representations depended on shortcut strength $\varphi _\text {train}$. For high $|\varphi _\text {train}|$, device dependence increased toward deeper layers. For low $|\varphi _\text {train}|$, device dependence decreased whereas health status dependence became more prominent, eventually surpassing device dependence in the deeper layers (Supplementary Figs. S5, S7 and S8). Final-layer $\textrm{nHSIC}_D$ rose monotonically with $|\varphi _\text {train}|$, dominating $\textrm{nHSIC}_Y$ for $|\varphi _\text {train}>0.25|$ (Supplementary Fig. S6).

## Discussion

This study provides the first systematic investigation of hardware-induced confounding factors in photoacoustic imaging. Our results demonstrate that hardware-induced confounding in photoacoustic imaging is not a marginal technical artifact, but a fundamental and largely underestimated challenge for deep-learning–based analysis.

Across four photoacoustic systems of the same model (MSOT Acuity Echo), we observed hardware-dependent variations, most notably complex parasitic noise patterns, sensor degradations, and membrane-related effects, which imprint distinct signatures on the reconstructed images, here referred to as device fingerprints.

We introduced a comprehensive correction pipeline tailored to minimize these device fingerprints, which substantially reduced visible device-specific signatures. However, despite those corrections, the remaining device-specific fingerprints were still sufficient for DL models to identify device identity with near-perfect accuracy. Notably, DL-based device detection remained clearly above chance even for small image patches, indicating that residual device cues were spatially distributed. This shows that even extensive preprocessing is insufficient to remove device-specific information and that device fingerprinting in PAI is stronger and harder to suppress than one might intuitively expect.

This unexpected robustness of device fingerprints has critical implications: When device instance correlates with health labels during training, disease classification models engage in shortcut learning, focusing on these device-specific cues rather than pathology and thereby producing biased and clinically misleading predictions. Representation-level analyses confirm this shortcut mechanism, with stronger device shortcuts in training sets leading to final representations dominated by device information over health status.

These findings extend the growing body of evidence on hardware-related bias in medical imaging AI. Previous work in MRI, CT, and radiography has shown that scanner-specific hardware and acquisition protocols can act as confounders and induce shortcut learning, even when such differences appear subtle to human observers^2,4,8^. While the general concept that confounding can induce shortcut learning is well established, a systematic investigation of analogous effects in PAI has been lacking, and it has therefore remained unclear whether they are sufficiently persistent and pronounced to meaningfully bias downstream prediction models in realistic multi-device and multicenter settings.

The striking insight that such effects occur even within a single model line of PAI devices underscores the susceptibility of emerging modalities where device designs and calibration protocols are still evolving. The observation that subtle device fingerprints were sufficient to bias PAD classifiers challenges the widespread assumption that pooling data from identical device instances of the same model is methodologically safe. It further illustrates that robustness and fairness cannot be guaranteed simply by balancing the marginal distribution of the target labels. As long as the joint distribution of the confounder (e.g., device instance) and target variable (e.g., health status) remains skewed across both training and test sets, model biases can go unnoticed. Models may achieve seemingly excellent internal performance where this joint distribution remains stable, thus where the device–health correlation $\varphi$ does not shift between training and test data. However, they tend to generalize poorly to data in which the joint distribution and therefore the confounder–target covariate structure differs. In this sense, the practical value of our study lies not only in confirming a shortcut-learning mechanism, but in identifying a concrete and easily overlooked source of hidden bias that is highly relevant for multi-device PAI studies, particularly in multicenter settings and future clinical deployment. Although the settings with $|\varphi |=1$ served only as a boundary case rather than a typical clinical scenario, the observed bias was not confined to this extreme, but was already evident at $|\varphi _\text {train}|=0.25$ and increased progressively with $|\varphi _\text {train}|$ across the evaluated correlation range. In multicenter PAI studies, unless recruitment is explicitly balanced across device and health status strata, non-negligible device–health correlations may arise through site- or cohort-specific prevalence differences between populations measured with different devices or, particularly for small datasets, even through statistical fluctuations in dataset composition.

Compared with patient sex, which has recently been identified as a confounding factor in PAI^17^, device identity emerged as an even stronger source of bias in this dataset. Concretely, the normalized Hilbert-Schmidt Independence Criterion in the PCA embedding was substantially higher for device identity ($\textrm{nHSIC}_D=0.440$ before correction, 0.189 after correction) than for sex ($\textrm{nHSIC}_\textrm{sex}=0.067$ and 0.103, respectively), indicating that device-related structure dominates over sex-related confounding in our data. This underscores that hardware-related fingerprints constitute one of the most relevant hidden confounders in clinical PAI.

Nevertheless, several limitations and open questions remain. The current findings of shortcut learning were derived from two MSOT device versions (non-CE/CE), meaning that part of the observed shift might reflect version-specific hardware differences rather than instance-level variability alone (see Supplementary Tab. S1). While still of the same model, replicating the experiments with device instances of the identical version should be performed to confirm the generality of the effect.

While our analysis focused on shortcut learning arising from differences between two device instances, many studies develop models within a single-device setting. However, even in this scenario, within-device factors may induce shortcut learning. In particular, temporal shifts caused by sensor degradation, drift in membrane characteristics, software updates, or hardware maintenance are plausible in practice and may become spuriously correlated with disease labels. This risk is especially pronounced when cohorts are enrolled sequentially rather than in parallel, because time-dependent device fingerprints can then become entangled with the target labels and be exploited by the model as shortcuts.

Collecting data from a large number of devices and under diverse acquisition conditions may reduce the risk of shortcut learning by making any single device fingerprint less predictive. However, this depends on sufficient sample size and balanced representation across devices, acquisition conditions and target labels; otherwise, residual spurious correlations and domain-shift effects may persist.

Beyond device-level confounders, the literature suggests that additional factors such as patient-related factors (demographic attributes like skin color^16^ and sex^17^, body mass index^26,27^, medication^28^ and blood hemoglobin variations^29^) or further acquisition-related factors like operator variability^30^ or room temperature^31^ may also cause bias, warranting systematic investigation in future work.

In addition to the device-specific artifacts analysed here, further acquisition-related signals are known to exist in MSOT devices, such as first arriving signals and their reflections, as investigated by Longo^32^. These effects can generate strong device-specific responses outside the standard field of view. Because they fall largely outside the imaging region considered in this work, they were not the primary focus of the present analysis, but may further reinforce device-specific fingerprints in other settings.

Our analysis was limited to image data reconstructed with one reconstruction algorithm (delay-and-sum algorithm). Alternative algorithms (deep learning-based, iterative) might exhibit reduced device fingerprinting and consequently, device fingerprint detectability may depend on the reconstruction method used.

The risk of shortcut learning dominance depends not only on $\varphi _\text {train}$ but also on the relative difficulty of the downstream task versus confounder detectability. In our data, device instance classification was near-perfect ($\textrm{AUROC} = 1.0$ for full FOV image data), whereas PAD classification was markedly harder ($\textrm{AUROC}\approx 0.77$), making models more prone to exploiting the easily-learnable device shortcut even at relatively low $|\varphi _\text {train}|$. In other multicenter studies with subtler device fingerprints or simpler clinical tasks, higher $|\varphi _\text {train}|$ values might be less critical.

The analyses in this paper focused on one convolutional neural network (CNN) architecture (EfficientNetV2^33^) for the main experiments. More generally, bias and shortcut learning are general issues across machine learning (ML) models, including state-of-the-art transformers and vision–language models^34,35^. Supporting this claim, repeating experiments with transformer-based SwinV2-T architecture^36^ confirmed consistent shortcut-learning patterns (Supplementary Figs. S9-11). Thus, the issues observed here are likely to extend beyond the specific model choice.

Methodologically, bias due to shortcut learning can be addressed using three principal approaches 1) reducing detectability by suppressing confounder-related features in the data, 2) reducing utility of the confounder by actively breaking spurious correlations between the confounder and the target variable in the training data, and 3) training bias-aware models to promote invariance to confounders and disentangle task-relevant signals from confounder-related features. Here we adopt the terminology of “detectability” and “utility” as proposed by Pavlak and Drenkow et al.^8,37^. As shown in Fig. 4, device-specific fingerprints remain highly detectable even after correction, while our subsequent experiments (Fig. 5) show that these detectable signals only become a harmful shortcut when dataset design assigns them predictive utility via device–disease correlations.

1. Reducing detectability: While the applied corrections reduced some device fingerprints, no preprocessing method we tested removed them entirely. The high device detectability after correction (Fig. 4) indicates the limitations of the current correction pipeline. In particular, residual complex parasitic noise artifacts likely persisted after the correction, as suggested by the arc-shaped Grad-CAM pattern at $\varphi _\text {train}=-1.0$. Improving the correction pipeline to reduce device detectability is part of future work. Advanced post-processing methods may further suppress device-related fingerprints. For example, the approach by Dehner et al.^38^ targets complex parasitic noise, but requires dedicated in aqua data for each device. In addition, the current pipeline does not explicitly model inter-device membrane differences. The observed variations in membrane SNR and membrane characteristics across devices (Fig. 2a) suggest that differences in membrane optical properties may affect light transmission and thus the fluence distribution in tissue, which could contribute to residual device-dependent variation in the measured signals. Moreover, the depth alignment step standardizes skin depth by repositioning the FOV, but does not correct fluence variations from probe filling differences, which can also cause spectral coloring. Future work could therefore also explore device-specific fluence modeling, for example by developing a digital device twin that incorporates membrane variations and estimating device-specific fluence via Monte Carlo simulations. Addressing device-related confounding might also necessitate advances in hardware design. In addition, establishing standardized calibration protocols and creating open benchmark datasets that span multiple devices and centers will be essential to reliably assess and enhance cross-device generalization.
2. Reducing utility: In our experiments, reducing utility by undersampling to achieve balanced device–health relations (such as setting $\varphi _\text {train}$=0) successfully produced fair models. However, this approach comes with tradeoffs: undersampling reduces training variability, which in data-limited settings can make model predictions noisier and less reliable. Advances in generative modeling, such as counterfactual data synthesis, may offer solutions by augmenting underrepresented strata and minimizing spurious confounder–target associations^39,40^. At the same time, reducing utility does not ensure that device-related information is no longer encoded in the learned representations. Even at $\varphi _\text {train}=0$, device-related information remained comparatively pronounced in early and intermediate layers, whereas health-related information became more prominent only in deeper layers (see Supplementary Fig. S5). Although this residual encoding did not appear to substantially affect the final decision function in our setting, it may still affect representation quality, which would become especially critical when such models are reused as generic feature encoders for other tasks.
3. Training bias-aware models: Future work could explore methods that promote model invariance to confounders like device fingerprints and disentangle pathology from hardware artifacts. This can include integrating knowledge of device identity into model training via empirical risk minimization strategies penalizing poor subgroup performance through subgroup reweighting^41,42^, conditional prevalence adjustment for anti-causal tasks^43^, adversarial debiasing^44^, physics-informed data augmentation^45^, physics-driven data generation strategies (e.g., via simulations)^46^, disentangled representation learning^47^, and counterfactual contrastive learning^48,49^, or subgroup-label-free methods needing no device identity information like self-identified hard example upweighting (Just Train Twice)^50^.

For efficient systematic bias detection, we introduced $\varphi$-dependent metrics such as balanced accuracy BA($\varphi$), AUROC($\varphi$), offering valuable tools for bias detection under covariate shift beyond PAI. Without knowing a test set’s underlying $\varphi$, model generalization can be easily overestimated, masking shortcut learning and biased predictions. Thus, $\varphi$-dependent evaluation and subgroup analysis facilitates bias-aware model validation.

In conclusion, this study shows that device-induced confounding is a critical obstacle for developing reliable and generalizable AI models in PAI. Even minor hardware variability within a single model family can imprint strong fingerprints that distort downstream predictions. Recognizing and addressing these effects is essential for confounder-aware modelling and for the design of harmonized acquisition and calibration protocols. Such efforts will be key to enabling robust multicenter PAI studies, identifying truly clinically meaningful applications, and ultimately ensuring that AI supported diagnosis in PAI is both trustworthy and fair.

## Materials and methods

Following ethics approval, this section presents the datasets analyzed and the experimental design addressing the three research questions.

### Ethics

This study was conducted in accordance with the principles of the Declaration of Helsinki and was approved by the ethics committee of the Medical Faculty of the University of Erlangen–Nuremberg. The clinical investigations were registered at ClinicalTrials.gov under the identifiers NCT04641091, NCT05373927, and NCT05773534. All participants provided written informed consent prior to inclusion in the study.

### Datasets

Two complementary datasets were analyzed in this work: a water bath dataset for characterizing device-specific image features and an in vivo dataset comprising healthy volunteers and patients with PAD. These are referred to as the in aqua (water bath) and in vivo datasets, respectively.

#### Imaging devices

For photoacoustic (PA) image acquisition, four PAI device instances of the type MSOT Acuity Echo (iThera Medical GmbH, Munich, Germany) were used (see Supplementary Tab. S1). All device systems employed an Nd:YAG laser with a tunable wavelength range of $660$ to $1300\,\textrm{nm}$, a pulse energy of $30\,\textrm{mJ}$, a repetition rate of $25\,\textrm{Hz}$, and a pulse duration between $4$ and $10\,\textrm{ns}$. Each system was equipped with an arc-shaped one-dimensional ultrasonic detector array comprising 256 elements with a center frequency of $4\,\textrm{MHz}$ ($60\,\%$ bandwidth).

#### In aqua dataset

For the in aqua dataset, the imaging probe was rigidly mounted above a glass water tank using a steel arm to ensure a stable and reproducible acquisition geometry. The dimensions of the glass cylinder were chosen such that acoustic waves did not reach and reflect from the glass walls within the acquisition time. For devices 2, 3, and 4, more than 45,000 frames were acquired in water over a wavelength range of $660$ to $1300\,\textrm{nm}$.

#### In vivo dataset

The in vivo PAD dataset included data from three clinical studies^51–53^ performed at the Department of Vascular Surgery, University Hospital Erlangen, Germany, using devices 1 and 2, for which complete metadata (age, sex, and device identity) were available. Recruitment details and diagnostic criteria are available in the corresponding clinical trial registrations (ClinicalTrials.gov: NCT04641091, NCT05373927, and NCT05773534). In these studies, calf measurements were obtained before and after exercise; for the present analysis, only 2D images acquired before exercise were used in order to exclude exercise-related effects. Moreover, only PAD patients with intermittent claudication (Fontaine stage IIa or IIb^54^) were included to reduce inter-study heterogeneity. For the present analysis, we used multispectral images at $730 ,\ 760 ,\ 800 ,\ 850 ,\ 930$, and $1300\,\textrm{nm}$, corresponding to the wavelength range with the greatest overlap across the three studies. In total, the dataset comprised 142 healthy volunteers (33 male/34 female imaged with device 1 and 32 male/41 female with device 2) and 153 PAD patients (54 male/27 female imaged with device 1 and 45 male/26 female with device 2), yielding 295 subjects overall with a mean age of 66 and 72 for healthy and diseased patients, respectively.

#### Data preprocessing and image reconstruction

Both the in aqua and in vivo datasets were reconstructed and preprocessed using the toolkit for Simulation and Image Processing for Photonics and Acoustics (SIMPA)^46^. The raw time-series signals were first corrected for laser pulse energy and filtered with a band-pass filter based on a Tukey window with an alpha value of 0.5, a high-pass cutoff of $50\,\textrm{kHz}$, and a low-pass cutoff of $10\,\textrm{MHz}$. Delay-and-sum beamforming was applied with a voxel spacing of $0.1\,\textrm{mm}$ and a homogeneous speed of sound of $1540\, \textrm{m}\,\hbox {s}^{-1}$ to reconstruct the PA images. Subsequently, envelope detection was performed in the image domain using the Hilbert transform. Between laser energy correction and beamforming, optional correction procedures were applied for specific experiments; these methods are described in detail below (Signal correction methods).

### Experimental design

**Fig. 8:**
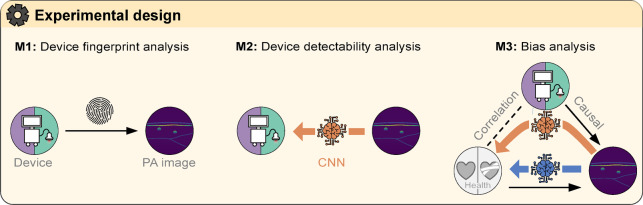
Conceptual overview of the experimental design. From left to right, the methodological contributions include: **M1** Device fingerprint analysis to identify which device-specific signatures are embedded in the photoacoustic (PA) image. **M2** Device detectability analysis to quantify the detectability of device instance identity in in vivo PA images, and **M3** bias analysis to assess whether these device fingerprints can bias deep learning models trained for disease diagnosis. Black arrows indicate causal relationships, while the dotted line represents a spurious correlation. Colored arrows denote convolutional neural networks (CNNs) that either rely on device-specific features (orange) or disease-related features (blue).

To demonstrate the existence of device-specific fingerprints and to illustrate the potential risks they pose for DL models that may inadvertently exploit them, we conducted three major experiments (Fig. 8).

#### Device fingerprint analysis

We selected device-specific fingerprints that (i) originate from principal physical subsystems of the PAI image-formation chain (illumination, acoustic coupling, US sensors and their readout electronics)^55^, and (ii) are known to induce structured artifacts in images^56^. These were characterized using the in aqua measurements capturing membrane properties, noise, and laser energy, and the in vivo data assessing sensor degradations. For readers interested in a comprehensive and detailed analysis of device fingerprints in MSOT data, they are encouraged to consult the master’s thesis of Bender^57^.

To quantify the membrane SNR, device-specific membrane masks were defined from the mean reconstructed $1210\,\textrm{nm}$ image, at which the membrane signal was most prominent. For each frame and wavelength, the membrane signal was extracted from these masks, corrected for the corresponding laser pulse energy, and related to the estimated image noise to obtain the SNR. Full implementation details are given in Supplementary Algorithm 1.

To quantify the thermal noise level of each sensor, we followed an approach analogous to Dehner et al., who, besides characterizing standard thermal noise, also termed the PAI-specific additive noise pattern induced by electromagnetic interference as complex parasitic noise^38^. Time-series segments free of complex parasitic noise were identified by applying a dedicated detection algorithm across all available image frames and wavelengths. Within these cropped thermal-noise-only regions, the Gaussianity of sensor-specific noise distributions was verified using Kolmogorov-Smirnov tests and Q-Q plot evaluation. The thermal noise level of each sensor was then determined by calculating the standard deviation across the time dimension, aggregated over all images acquired during the experiment.

To analyze the laser energy values, we extracted them directly from the MSOT device metadata associated with the in aqua measurements.

#### Device detectability analysis

To assess whether device-specific fingerprints are detectable in in vivo images, the in vivo dataset was analyzed both qualitatively and quantitatively. Qualitative analyses included visual comparison of representative images and low-dimensional embeddings obtained by PCA. Quantitatively, device predictability was evaluated through the classification performance of DL models trained to infer the imaging device from in vivo images.

**Principal component analysis** PCA was applied to the in vivo images in order to explore device-related structure in a model-agnostic manner. Prior to PCA, all images were z-score normalized so that each feature had zero mean and unit variance. For qualitative visualization, the data were embedded into the two-dimensional subspace spanned by the first two principal components (PCs), and points were color-coded by device to visually inspect device clustering before and after corrections. To further assess whether the observed structure was attributable only to device instance or also to disease-related variation, the embeddings were additionally color-coded by health status.

**Normalized Hilbert-Schmidt Independence Criterion (nHSIC)** To quantify the dependence between image representations and device instance and health status, we used a normalized variant of the Hilbert-Schmidt Independence Criterion (HSIC)^18,19^, which is given by

1 $$\begin{aligned} \textrm{HSIC}(\mathbf{X}, \mathbf{Z})&= \frac{1}{(n-1)^2}\operatorname {tr}(\mathbf{K}\mathbf{H}\mathbf{L}\mathbf{H}) \end{aligned}$$

for $\mathbf{X} \in \mathbb {R}^{n \times p}$ and $\mathbf{Z} \in \mathbb {R}^{n \times q}$. $\mathbf{K}$ and $\mathbf{L}$ are the kernel similarity matrices computed on $\mathbf{X}$ and $\mathbf{Z}$, respectively, and $\textbf{H} = \mathbf{I}_n - \frac{1}{n}\mathbf{1}\mathbf{1}^\top$ is the centering matrix. The normalized HSIC, also known as Centered Kernel Alignment (CKA), is then

2 $$\begin{aligned} \textrm{nHSIC}(\mathbf{X}, \mathbf{Z})&= \frac{\textrm{HSIC}(\mathbf{X}, \mathbf{Z})}{\sqrt{\textrm{HSIC}(\mathbf{X}, \mathbf{X})\,\textrm{HSIC}(\mathbf{Z}, \mathbf{Z})}}. \end{aligned}$$

Using linear kernels, $\mathbf{K} = \mathbf{X}\mathbf{X}^\top$ and $\mathbf{L} = \mathbf{Z}\mathbf{Z}^\top$, this reduces to

3 $$\begin{aligned} \textrm{nHSIC}(\mathbf{X}, \mathbf{Z})&= \frac{\left\Vert \mathbf{Z}_c^\top \mathbf{X}_c \right\Vert _F^2}{\left\Vert \mathbf{X}_c^\top \mathbf{X}_c \right\Vert _F \left\Vert \mathbf{Z}_c^\top \mathbf{Z}_c \right\Vert _F}, \end{aligned}$$

where $\mathbf{X}_c = \mathbf{H}\mathbf{X}$ and $\mathbf{Z}_c = \mathbf{H}\mathbf{Z}$ are the centered versions of $\mathbf{X}$ and $\mathbf{Z}$, and $\Vert \cdot \Vert _F$ denotes the Frobenius norm^20^.

In our analysis, $\mathbf{Z}$ corresponded either to the device labels $\mathbf{D} \in \mathbb {R}^{n \times 1}$ or the health status labels $\mathbf{Y} \in \mathbb {R}^{n \times 1}$, yielding the dependence measures $\textrm{nHSIC}_D$ and $\textrm{nHSIC}_Y$, respectively. For the PCA-based analysis, $\textrm{nHSIC}_D$ and $\textrm{nHSIC}_Y$ were computed on the principal components explaining $80\,\%$ of the total variance. Statistical significance was assessed using permutation tests with $1{,}000$ repetitions.

**Signal correction methods** To assess whether device fingerprints can be removed by plausible preprocessing and thereby evaluate the practical relevance of our findings, several signal correction procedures were implemented and integrated into the preprocessing pipeline. These methods were applied after laser energy correction and before beamforming reconstruction, and were designed to specifically target artifacts and noise patterns associated with individual devices. In addition to temporal averaging, which is widely used in PAI, and singular value decomposition (SVD)-based noise reduction as previously reported in the literature^38^, new correction strategies were introduced that, to the best of our knowledge, have not yet been described for clinical PAI. All correction methods are described briefly below in order of processing.

(i) **Early response sensor correction**: Temporally shifted sensor responses were corrected by aligning each affected signal to an interpolated reference derived from neighboring sensors using cross-correlation-based time-shift estimation. Full implementation details are provided in Supplementary Algorithm 3.
(ii) **SVD-based reduction of complex parasitic noise:** To reduce complex parasitic noise while preserving tissue signal, an SVD-based denoising approach was employed that operated only on the outer sensor bands (sensors $n \in \{1,\dots ,32\}$ and $n\in \{225,\dots ,256\}$), instead of all. Previous work has shown that SVD is effective for suppressing structured noise in preclinical PA systems^58,59^, but applying it uniformly across all sensors in the present clinical setting led to substantial attenuation of tissue signals. Restricting SVD to the outer bands provided a trade off between removing device specific parasitic noise and maintaining diagnostically relevant signal content.
(iii) **Temporal averaging:** Temporal averaging was applied as a conventional noise suppression technique^29,60^. For each wavelength, eight consecutive measurement frames were averaged to suppress thermal noise and residual complex parasitic noise and enhance SNR.
(iv) **Broken sensor interpolation:** Sensors that produced only noise and no discernible signal were classified as broken. Broken sensors were replaced by an interpolated signal derived from the nearest functioning left and right neighbors after temporal alignment. Details are given in Supplementary Algorithm 2.
(v) **Depth alignment:** Finally, a depth alignment procedure was applied to standardize the apparent skin position across devices. The skin depth was estimated by detecting the maximum superficial peak in the reconstructed signal. The reconstruction FOV was then redefined to map this skin surface to a consistent depth across all devices. This step minimized systematic depth offsets between device instances and enabled device-agnostic comparison of tissue structures with respect to depth.

**Deep-learning-based device classification** Torralba et al. proposed the idea of training models to predict the dataset identity in order to expose dataset-specific biases and termed this strategy “Name That Dataset”^61^. This concept can be generalized to any known confounder. In the present work, it was adapted to “Name That Device”, that is, a supervised classification task where the imaging device instance identity serves as the target label. Successful device prediction from in vivo images indicates that device-specific fingerprints are present and exploitable by discriminative models^8,37^.

*Train-test split* A fixed train-test split was defined once and used consistently throughout all experiments. The split was performed at the patient level to prevent data leakage, ensuring no subject appeared in both training and test cohorts. The test set was constructed to be balanced with respect to device, sex, and health status ($\varphi =0$, see Eq. (4)), yielding 64 subjects in the final test cohort. All remaining subjects formed the training pool from which five cross-validation folds were drawn.

*Robustness of device detectability* To investigate the robustness of device detectability on the corrected image data, models were trained and evaluated on datasets subjected to systematic spectral and spatial cropping. Spectrally, multispectral and single-wavelength inputs were compared to examine whether device prediction relies on higher wavelengths with lower SNR. Spatially, images were cropped to four different fields of view: full FOV ($24\, \textrm{mm} \times 36\, \textrm{mm}$), large patch ($16\, \textrm{mm} \times 24\, \textrm{mm}$), small patch ($6\, \textrm{mm} \times 6\, \textrm{mm}$), and mini patch ($3\, \textrm{mm} \times 3\, \textrm{mm}$). Since superficial pixels above the skin predominantly capture device-dependent signals like residual streaking artifacts, crops were progressively tightened to tissue-only regions to test detectability with reduced spatial context and less device-dependent artifacts. For patch ($6\,\textrm{mm}\times6\,\textrm{mm}$) and minipatch ($3\, \textrm{mm} \times 3\, \textrm{mm}$) settings, random patches were extracted from the tissue FOV during training to prevent overfitting on limited data volumes, while central patches were used deterministically during testing to ensure comparability and reproducibility across models.

*Model development and training* Device classification was performed using an EfficientNetV2-S architecture^33^. Published weights pretrained on ImageNet were imported via the corresponding implementation package^62^. Previous work on PA image analysis has demonstrated successful application of ImageNet-pretrained CNN models to PA images^17^, which motivated the choice of a pretrained convolutional backbone in this study. Our work also considered multispectral inputs with more than three wavelengths. To accommodate this, the first convolutional layer of EfficientNetV2 was modified to accept a variable number of input channels equal to the number of wavelengths, instead of the default three red, green, and blue (RGB) channels, by duplicating the original filter weights across channels. The final classification layer was replaced to output one logit per device class. No layers were frozen, and the entire network was fine-tuned end-to-end on the training data.

Binary cross entropy loss was used for optimization and the AdamW optimizer with decoupled weight decay was employed^63^. Hyperparameters (including learning rate, weight decay, and optimizer parameters) were tuned through a combination of manual exploration and systematic hyperparameter optimization using Hydra and Optuna^64,65^; the final hyperparameter settings are summarized in the Supplementary (Tab. S3 and S4).

A stratified fivefold Monte Carlo cross-validation scheme with maximally distinct validation sets was adopted on the training pool^66^. Within each device–health stratum, data were first randomly partitioned into five folds that were as equally sized as possible. For the *k*-th CV iteration, the *k*-th fold samples formed the core validation set. Additionally, samples were drawn from the $((k\bmod 5)+1)$-th fold until exactly 40 training samples remained per stratum. This ensured balanced representation without device–health correlations ($\varphi _\text {train}=0$) across folds while maximizing validation set distinction. This approach resulted in 160 subjects forming the training set and 71 subjects constituting the validation set for each fold. Common data augmentation strategies for medical image classification were applied, and their details are provided in the Supplementary (Tab. S3). After training, temperature scaling was performed on the validation sets, with the calibration objective reweighted so that each device–health stratum contributed equally to the calibration loss. Final predictions for the held-out samples of the test set were obtained by averaging the calibrated scores across the five folds to form the model ensemble output. Separate ensembles were trained for each combination of spectral condition (multispectral/monospectral) and field-of-view cropping.

*Evaluation of device detectability* Following current best practice recommendations from “Metrics Reloaded”^67^, device classification performance was evaluated using the AUROC and balanced accuracy (BA) as primary metrics. Confidence intervals for all metrics were obtained via bootstrap resampling of the fixed test set (1,000 bootstrap replicates, with stratification by device–health stratum).

#### Bias analysis for disease diagnosis

To assess how device detectability biases disease classification, we trained DL model ensembles on resampled subsets of the PAD dataset, systematically varying the spurious association between device identity and health status ($\varphi$). The disease classification model ensembles were then evaluated on a held-out test set and the balanced accuracy BA($\varphi$) as a function of $\varphi$, and AUROC($\varphi$) was determined spanning a full range of shortcut intensity, from $\varphi =-1.0$ to $\varphi =1.0$. Here, $\varphi$ denotes the phi coefficient, which was originally introduced by Pearson^21^ and formally defined for contingency tables by Cramér^22^. In this context, it quantifies the association between device instance and the health status, here termed the device–health correlation and given by

4 $$\begin{aligned} \varphi = \frac{N_{\text {1,diseased}} \, N_{\text {2,healthy}} \;-\; N_{\text {2,diseased}} \, N_{\text {1,healthy}}}{\sqrt{N_{\text {1}}\, N_{\text {2}}\, N_{\text {diseased}}\, N_{\text {healthy}}}}. \end{aligned}$$

Whereby $N_{D,Y}$ denotes the number of subjects with health status $Y\in \{\text {healthy},\text {diseased}\}$ measured on device instance $D\in \{1,2\}$. The marginal counts $N_Y$ refer to the total number of subjects with health status *Y* regardless of device *D*, and $N_D$ denotes the total number of subjects measured on device *D* irrespective of health status *Y*. Using these counts, $\varphi$ captures the degree of spurious correlation between health status and device, where

- $\varphi =-1.0$ corresponds to perfect negative correlation, i.e.: all diseased subjects originate from Device 2 and all healthy subjects from Device 1;
- $\varphi =0.0$ corresponds to no correlation: health status and device origin are statistically independent;
- and $\varphi =1.0$ corresponds to perfect positive correlation: all diseased subjects originate from Device 1 and all healthy subjects from Device 2.

**Data splitting and sampling** To systematically study shortcut learning arising from spurious device–health correlation, we generated validation sets and training datasets with predefined $\varphi$ values from the training pool. The same underlying training pool and test set as in the previous experiment were used.

Nine datasets were constructed for nine correlation levels, ranging from $\varphi =-1.0$ to $\varphi =1.0$ in increments of 0.25, and a separate ensemble of models was trained for each level. For every $\varphi$, five independent runs of stratified Monte Carlo sampling were performed, corresponding to five ensemble members. In each run, training sets were generated by sampling without replacement from the training pool within each device–health stratum (*D*,*Y*) such that the resulting counts $N_{D,Y}$ produced the desired target correlation $\varphi$. The total number of subjects per training set was kept fixed at 96, with equal overall health status balance and equal device representation enforced by setting $N_1=N_2=N_\text {healthy}=N_\text {diseased}=48$. All remaining subjects for a given $\varphi$ and run were assigned to the corresponding validation set. The resulting configurations of $N_{D,Y}$ across all correlation levels are summarized in the Supplementary (Tab. S5).

**Training and evaluation** In order to analyze whether DL models engage in shortcut learning overrelying on device-related features, we trained EfficientNetV2-S models for PAD classification on the corrected full FOV image data. Model training and optimization followed the general setup described for device classification, including hyperparameter tuning conducted with the same approach and tools. However, a more extensive hyperparameter search was needed to accommodate the increased complexity of the PAD classification task. The final hyperparameter settings are reported in the Supplementary (Tab. S3 and S6). For each $\varphi _\text {train}$ and each Monte Carlo run, training was performed on the corresponding resampled training set. Model calibration was conducted analogously to the device classification experiment using temperature scaling on the validation set, but the calibration loss was reweighted according to the device–health distribution induced by the corresponding $\varphi _\text {train}$ level to reduce effects of covariate shifts between training and validation. For each $\varphi _\text {train}$, the five calibrated models obtained from the independent Monte Carlo runs were combined into an ensemble by averaging their calibrated prediction scores on the test set.

For the evaluation of disease classification under varying device–health correlations, performance and fairness metrics were defined at the level of device–health subgroups and integrated into $\varphi$-dependent classification metrics. Device-specific specificity and sensitivity were computed as

5 $$\begin{aligned} \text {TNR}\big |_{d}&:= \mathbb {P}(\widehat{Y}\mathrel {\hspace{-1.66656pt}=\hspace{-1.66656pt}}\text {healthy}\mid Y^*\mathrel {\hspace{-1.66656pt}=\hspace{-1.66656pt}}\text {healthy},D=d) \approx \frac{\text {TN}\big |_d}{\text {TN}\big |_d+\text {FP}\big |_d}\text {, and} \end{aligned}$$

6 $$\begin{aligned} \text {TPR}\big |_{d}&:= \mathbb {P}(\widehat{Y}\mathrel {\hspace{-1.66656pt}=\hspace{-1.66656pt}}\text {diseased}\mid Y^*\mathrel {\hspace{-1.66656pt}=\hspace{-1.66656pt}}\text {diseased},D=d) \approx \frac{\text {TP}\big |_d}{\text {TP}\big |_d+\text {FN}\big |_d}. \end{aligned}$$

For each device $d\in \{1,2\}$, where $Y^*\in \{\text {healthy},\text {diseased})$ denotes the reference health status and $\widehat{Y}$ the model prediction.

Under the assumption that in the test set the marginal prevalence of devices and health status is fixed and identical across all considered $\varphi$ values, the overall sensitivity and specificity of a dataset with four strata ($Y^*$,$D$) can be written as simple convex combinations of the subgroup quantities. In particular, the $\varphi$-dependent specificity and sensitivity are

7 $$\begin{aligned} \text {TNR}(\varphi )&= \frac{1-\varphi }{2} \text {TNR}\big |_{1} + \frac{1+\varphi }{2} \text {TNR}\big |_{2} \text {, and} \end{aligned}$$

8 $$\begin{aligned} \text {TPR}(\varphi )&= \frac{1+\varphi }{2} \text {TPR}\big |_{1} + \frac{1-\varphi }{2}\text {TPR}\big |_{2}, \end{aligned}$$

with the derivation provided in the Supplementary (Lemma 2). The corresponding $\varphi$-dependent balanced accuracy is then defined as

9 $$\begin{aligned} \text {BA}(\varphi ) = \frac{\text {TNR}(\varphi )+\text {TPR}(\varphi )}{2}, \end{aligned}$$

and an expression for a $\varphi$-dependent AUROC is given by

10 $$\begin{aligned} \textrm{AUROC}(\varphi ) = \frac{1}{8} \sum _{j=0}^{|C_{\text {thr}}|-1} \Big \{ \,&\big [ (1+\varphi )(\textrm{TPR}_{j}\big |_{1} + \textrm{TPR}_{j+1}\big |_{1}) + (1-\varphi )(\textrm{TPR}_{j}\big |_{2} + \textrm{TPR}_{j+1}\big |_{2}) \big ] \end{aligned}$$

11 $$\begin{aligned}&\cdot \big [ (1-\varphi )(\textrm{TNR}_{j}\big |_{1} - \textrm{TNR}_{j+1}\big |_{1}) + (1+\varphi )(\textrm{TNR}_{j}\big |_{2} - \textrm{TNR}_{j+1}\big |_{2}) \big ] \Big \}, \end{aligned}$$

and derived in the Supplementary (Lemma 3). Here, $C_\text {thr}$ denotes the ordered set of classification thresholds applied to the predicted disease probabilities on the test set $\{\hat{\mathbb {P}}(Y_i=1\mid x_i)\mid \forall x_i \in X_\text {test}\}$. For each device instance $d\in \{1,2\}$ and each threshold $c_\text {thr}^{j} \in C_\text {thr}$, $\text {TNR}_j\big |_d$ and $\text {TPR}_j\big |_d$ denote the corresponding true negative and true positive rates, respectively. The index *j* enumerates adjacent thresholds, so the sum implements a trapezoidal approximation of the $\varphi$-dependent receiver operating characteristic (ROC) curve by aggregating the areas between successive ROC points.

Model fairness was assessed using the separation notion ($\widehat{Y}\perp D|Y^*)$, which requires, given the true health status, that the predicted health status is independent of the device. Violations of separation were quantified by the disparity in specificity across devices

12 $$\begin{aligned} \Delta _\text {TNR}:= \text {TNR}\big |_1 - \text {TNR}\big |_2, \end{aligned}$$

and the sensitivity across devices

13 $$\begin{aligned} \Delta _\text {TPR}:= \text {TPR}\big |_1 - \text {TPR}\big |_2. \end{aligned}$$

Both $\Delta _\text {TNR}$ and $\Delta _\text {TPR}$ lie in the interval $[-1,1]$, with values near the boundaries indicating strong device dependence in the model’s predictions. In particular, $\Delta _\text {TNR}\approx -1$ together with $\Delta _\text {TPR}\approx 1$ implies model tends to predict healthy labels predominantly for measurements from device 2 and diseased labels predominantly for device 1. Conversely $\Delta _\text {TNR}\approx 1$ and $\Delta _\text {TPR}\approx -1$ indicate the opposite pattern. Such asymmetric prediction behaviour is indicative of the model’s exploitation of spurious device–health correlations present in the training data, leading to a reliance on device-specific rather than disease-related features.

Given a single fixed test set, this formulation allows analytical computation of $\text {BA}(\varphi )$, $\text {AUROC}(\varphi )$, $\Delta _\text {TNR}$, and $\Delta _\text {TPR}$ for all $\varphi \in [-1,1]$, once the subgroup performances $\text {TNR}\big |_d$ and $\text {TPR}\big |_d$ have been estimated. To quantify model uncertainty due to the test set variability, $n=1000$ stratified bootstrap replicates of the test set were generated, each time sampling 16 subjects per device–health subgroup and recomputing subgroup accuracies as well as $\Delta \text {TNR}$, $\Delta \text {TPR}$, $\text {BA}(\varphi )$, and $\text {AUROC}(\varphi )$ over the full range from $\varphi =-1.0$ to $\varphi =1.0$.

**Explainability** To interpret which image regions drove the disease classifier’s predictions, we employed the Grad-CAM variant HiResCAM via the PyTorch Grad-CAM package^68^. HiResCAM is a generalization of Grad-CAM^24,25^: instead of applying global average pooling, each feature map is weighted elementwise by its gradient before summation across channels. Grad-CAM maps were computed for all samples in the test set to obtain class-specific attribution patterns.

For each trained ensemble, Grad-CAMs were first generated for every individual ensemble member and then averaged to produce a single ensemble-level attribution map. This ensemble Grad-CAM provides a more robust and noise-reduced visualization of the model’s predictive focus than individual maps, and was used for all subsequent qualitative analyses.

**Latent space analysis** Latent feature encodings were extracted from the balanced 64-sample test set for each disease-classifier ensemble member, each $\varphi _\text {train}$ setting, and nine network locations of EfficientNetV2 (stages 0–7 and pre-logits). For quantitative analysis, we computed normalized $\textrm{nHSIC}_D$ and $\textrm{nHSIC}_Y$ between the latent encodings and the corresponding attribute labels at each network location, including the final layer. For qualitative visualization, we selected one representative intermediate layer (stage 5) and the final pre-logit layer. The corresponding encodings were z-score normalized feature-wise and projected onto the first two PCs, and the resulting embeddings were color-coded by either device instance or health status.

## Usage of large language models

Large Language Models (LLMs) were used to enhance the readability and linguistic quality of the manuscript. Following this, all content was reviewed and edited as necessary. The authors take full responsibility for the final published version.

## Supplementary information

Supplementary Information.

## Data Availability

De-identified individual participant data are not publicly accessible due to ethical constraints. However, the in aqua dataset can be obtained upon reasonable request by contacting the corresponding authors via email. Access to the data is limited exclusively to research purposes.
