# Supplementary material for: Photoacoustic device fingerprints induce bias in deep learning models
Source: Sci Rep. 2026 Jun 13;16:18695. doi: 10.1038/s41598-026-53468-6 (PMC13272909; doi:10.1038/s41598-026-53468-6)
Supplement: Supplementary file 1 — Supplementary Information. [file 41598_2026_53468_MOESM1_ESM.pdf]

Supplementary: Photoacoustic device fingerprints induce bias in deep learning models

Christoph J. Bender[1,2,*,†], **Marcel Knopp**[1,3,†], **Niklas Holzwarth**[1,3], **Tom Rix**[1,3], **Jan-Hinrich Nölke**[1,3], **Kris K. Dreher**[1,4,5,6], **Yi Li**[7], **Julius Kempf**[7], **Milenko Caranovic**[7], **Fabian Schneider**[1,8,9], **Melanie Schellenberg**[1,10], **Leonie Boland**[1,3], **Briain Haney**[7], **Ferdinand Knieling**[11], **Ulrich Rother**[7], **Alexander Seitel**[1,12,‡], **and Lena Maier-Hein**[1,2,3,6,12,13,14,15,*,‡]

[1]German Cancer Research Center (DKFZ) Heidelberg, Division of Intelligent Medical Systems (IMSY), Heidelberg, Germany
[2]Medical Faculty, Heidelberg University, Heidelberg, Germany
[3]Faculty of Mathematics and Computer Science, Heidelberg University, Heidelberg, Germany
[4]Faculty of Physics and Astronomy, Heidelberg University, Heidelberg, Germany
[5]now with German Cancer Research Center (DKFZ) Heidelberg, Division of Medical Image Computing (MIC), Heidelberg, Germany
[6]Helmholtz Imaging, German Cancer Research Center (DKFZ), Heidelberg, Germany
[7]Department of Vascular Surgery, University Hospital Erlangen, Friedrich-Alexander-Universität Erlangen-Nürnberg (FAU), Erlangen, Germany
[8]now with Institute of Biological and Medical Imaging, Helmholtz Zentrum München, Neuherberg, Germany
[9]now with Chair of Biological Imaging, Central Institute for Translational Cancer Research (TranslaTUM), School of Medicine and Health, Technical University of Munich, Munich, Germany
[10]now with Memorial Sloan Kettering Cancer Center, Department of Medical Physics, New York, USA
[11]Department of Pediatrics and Adolescent Medicine, University Hospital Erlangen, Friedrich-Alexander-Universität Erlangen-Nürnberg (FAU), Erlangen, Germany
[12]National Center for Tumor Diseases (NCT), NCT Heidelberg, a partnership between DKFZ and University Hospital Heidelberg, Heidelberg, Germany
[13]Heidelberg University Hospital, Surgical Clinic, Surgical AI Research Group, Heidelberg, Germany
[14]HIDSS4Health – Helmholtz Information and Data Science School for Health, Karlsruhe/Heidelberg, Germany
[15]Mohamed Bin Zayed University of Artificial Intelligence (MBZUAI), Abu Dhabi, UAE
[*]Email: christophjulien.bender@dkfz-heidelberg.de, l.maier-hein@dkfz.de
[†]These authors contributed equally to this work and share first authorship.
[‡]These authors shared equal leadership in this work.

1 Device characteristics

Device characteristics	Device 1	Device 2	Device 3	Device 4
Model	MSOT Acuity Echo	MSOT Acuity Echo	MSOT Acuity Echo	MSOT Acuity Echo
Version	Non-CE	CE	Non-CE	Non-CE
Transducer coating	Standard	Orange	Standard	Orange
Probe membrane	Highly scattering	More transparent	Highly scattering	More transparent
First arriving signal	✓	✗	✓	✗
Thermal noise	N/A	● ● ●	● ● ○	● ● ○
Complex parasitic noise	● ● ●	● ○ ○	● ● ●	○ ○ ○
Broken sensors	4 / 256	None	4 / 256	1 / 256
Early-response sensors	None	32 / 256	None	None
Laser energy level	N/A	● ● ●	● ● ○	● ● ○

Table S1. Overview of hardware specifications for the four photoacoustic devices. Devices differ in regulatory status (CE marking) and hardware generation. Newer devices 2 and 4 featured upgraded laser shielding to reduce complex parasitic noise in data aquisition modules, yet device-specific artifacts persisted. Filled circles indicate a more pronounced effect.

2 Model training details

2.1 Device detection models

	Device 1		Device 2	
φ_{train}	$N_{1,\text{healthy}}$	$N_{1,\text{diseased}}$	$N_{2,\text{healthy}}$	$N_{2,\text{diseased}}$
0.0	40	40	40	40

Table S2. Device-health subgroup counts $N_{D,Y}$ for balanced training set ($\varphi_{\mathbf{train}} = 0.0$). The total number of training samples was set to be $N_{\text{train}}^{(\text{tot})} = 160$. All device detection models were trained on data without any spurious device–health correlation.

**Hyperparameter category**	**Parameter**	**Value**
Model	Architecture	EfficientNetV2-S
	Weight initialization	ImageNet-pretrained weights
	Ensemble size	5 models
	Dropout rate (classification head)	$\mathbb{P}_{\text{dropout}} = 0.2$
Optimization	Number of epochs	50
	Optimizer	AdamW ($\beta_1 = 0.9$, $\beta_2 = 0.999$, weight decay $\lambda_{\text{AdamW}} = 0.01$)
	Learning rate	see Tab. S4
	Learning rate scheduler	Stepsize scheduler (every 10th epoch: $\eta \leftarrow \frac{1}{2}\eta$)
	Batch size	see Tab. S4
Data normalization and augmentation	Data scaling	$x_i \leftarrow \log(x_i + 1)$
	Normalization	Imagewise min-max (after all augmentation transformations)
	Gaussian noise augmentation	see Tab. S4
	Random flipping	horizontal with $\mathbb{P}_{\text{flipping horiz.}} = 0.5$
	Random affine transformation	$\theta \in [-3°, 3°]$, $\tau \in [-0.02, 0.02]$, $s \in [0.95, 1.05]$, $\phi \in [-3°, 3°; -1°, 1°]$

Table S3. Final hyperparameters used in all device detection experiments. Manual hyperparameter tuning was done to determine the listed hyperparameters. During training, each epoch consisted of 5 mini-batches randomly sampled with replacement from the training set. $\mathbb{P}_{\text{flipping horiz.}}$ denotes flip probability. Parameter ranges $[a, b]$ indicate uniform random sampling between bounds a and b. Here, θ denotes the rotation angle in degrees, τ the translation as fractional shift per axis, s the scale factor, and ϕ the shear angles in degrees (horizontal; vertical). Training was conducted for 50 epochs without early stopping, which was sufficient for the validation loss to converge across all settings.

**Spectral setting**	**Spatial setting**	**Batch size B**	**Learning rate η**	**Gaussian noise augmentation ε**
Multispectral	Full Image	16	0.0005	0
	Tissue Tile	32	0.0001	0.0005
	Patch	16	0.001	0.0005
	Minipatch	16	0.001	0.0005
$\lambda = 800$	Full image	32	0.0005	0.0005
	Tissue Tile	32	0.001	0
	Patch	16	0.001	0.0005
	Minipatch	16	0.001	0

Table S4. Optimized training hyperparameters across spectral and spatial settings. Automated hyperparameter optimization of batch size B, learning rate η, and Gaussian noise standard deviation ε was performed separately for each spectral and spatial configuration. Using Hydra with Optuna's TPESampler ($n_{\text{trials}} = 15$, $n_{\text{startup\_trials}} = 5$), optimal hyperparameters were identified by minimizing a weighted combination of validation loss, balanced accuracy (BA), and area under receiver operating characteristic curve (AUROC), each averaged over the last five epochs.

2.2 Disease diagnosis models

	Device 1		Device 2	
φ_{train}	$N_{1,\text{healthy}}$	$N_{1,\text{diseased}}$	$N_{2,\text{healthy}}$	$N_{2,\text{diseased}}$
-1.0	48	0	0	48
-0.75	42	6	6	42
-0.5	36	12	12	36
-0.25	30	18	18	30
0.0	24	24	24	24
0.25	18	30	30	18
0.5	12	36	36	12
0.75	6	42	42	6
1.0	0	48	48	0

Table S5. Device-health subgroup counts $N_{D,Y}$ for training sets across φ_{train} levels. For each φ_{train}-setting the total number of training samples was the same, $N^{(\text{tot})}_{\text{train}} = 96$. The (D,Y)-strata sizes satisfy the φ_{train} definition in Eq. (14) while maintaining balanced device and health marginals $N_1 = N_2 = N_{\text{diseased}} = N_{\text{healthy}} = 48$.

**Parameter**	**Value**
Dropout rate (classification head)	$\mathbb{P}_{\text{dropout}} = 0.24$
Batch size	$B = 32$
Learning rate	$\eta = 0.0007$
Gaussian noise augmentation	$\varepsilon = 0.0005$

Table S6. Optimized hyperparameters for disease diagnosis models. Most hyperparameters followed those in Table S3, except for dropout rate, batch size, learning rate, and Gaussian noise standard deviation. Extensive hyperparameter optimization ($n_{\text{trials}} = 100$, $n_{\text{startup\_trials}} = 10$) was performed exclusively for the $\varphi_{\text{train}} = 0.0$ setting using Optuna's TPESampler. Optimization targeted a weighted sum of validation BA($\varphi_{\text{test}} = 0.0$) and AUROC across device-health strata. These optimal hyperparameters (Table S6) were then applied to all φ_{train} settings, as other configurations exhibited substantial covariate shift between training and validation sets. During training, each epoch processed all training samples exactly once. Mini-batches maintained fixed φ_{train} by proportionally sampling from device-health strata without replacement.

3 Further results

3.1 Photoacoustic systems embed device-specific fingerprints

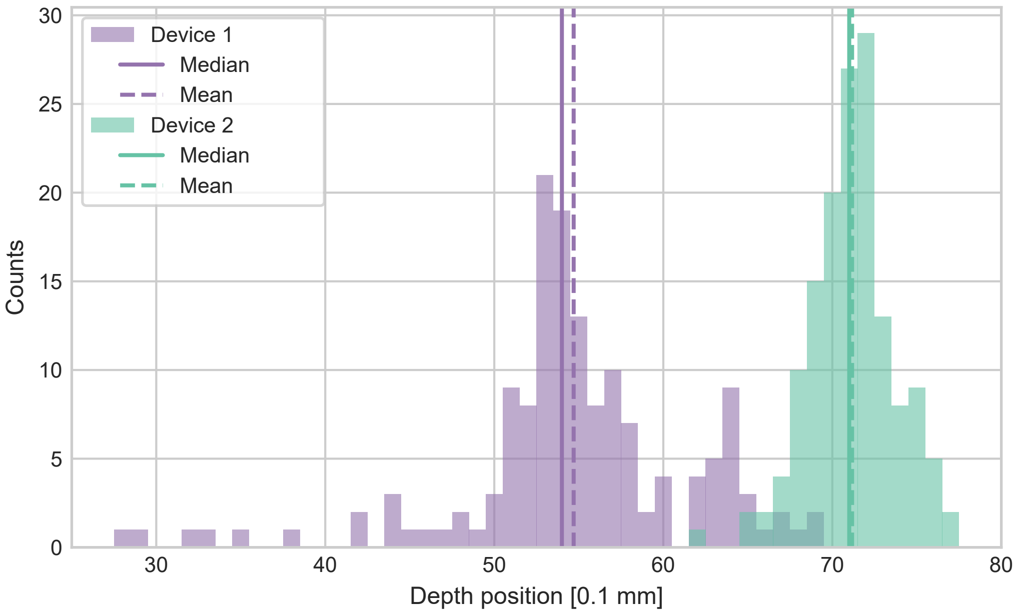

Figure S1. Device-specific skin depth positioning contributes to fingerprints. Skin depths were quantified on images with all presented signal corrections except depth alignment, revealing a pronounced offset. Skin position was determined by row-wise column averaging followed by argmax detection at $\lambda = 730\,\text{nm}$. The skin appears approximately 1.6 mm or 16 pixels deeper for Device 2, which has median and mean depths of 7.1 mm, than for Device 1, which has median depth 5.4 mm and mean depth 5.5 mm.

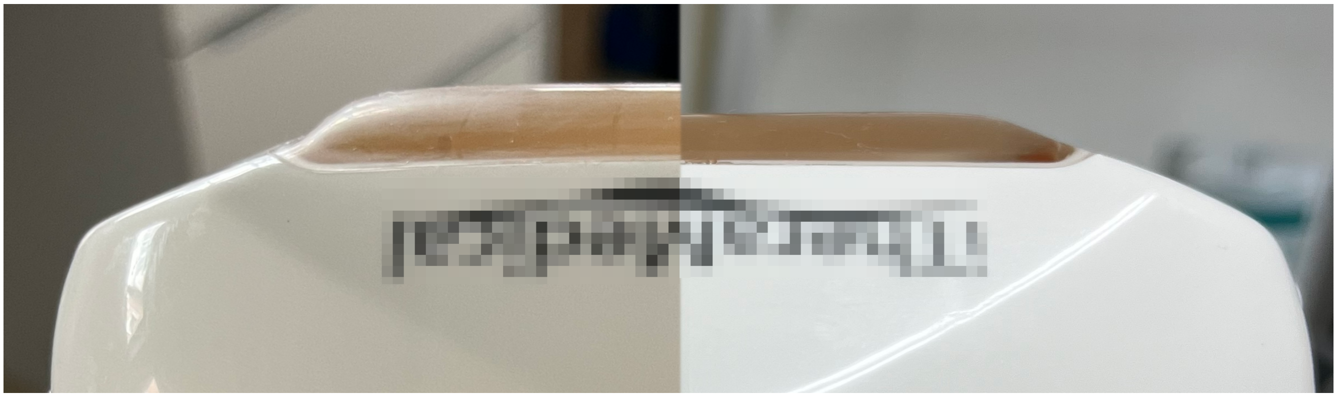

Figure S2. Membrane filling inconsistencies cause positional offsets between devices. In a manufacturer-provided example using two additional MSOT Acuity Echo probes, incomplete or uneven membrane transducer filling was associated with an approximately 3 mm positional shift of the membrane position relative to the imaging plane. For comparison, the in vivo dataset in Fig. S1 showed a smaller between-device skin-depth offset of approximately 1.6 mm. This observation suggests that membrane filling differences are a plausible contributor to the positional offsets observed in the in vivo dataset between devices 1 and 2 and can potentially be further amplified by operator-dependent pressure differences. Beyond inconsistent filling procedures, membrane properties are susceptible to temporal changes such as scratches or contamination, which can generate further systematic image artifacts[1]. Photo courtesy of Braden Eliason (iThera Medical GmbH).

3.2 Disease classification models can exhibit shortcut learning due to overreliance on device fingerprints rather than disease-related features

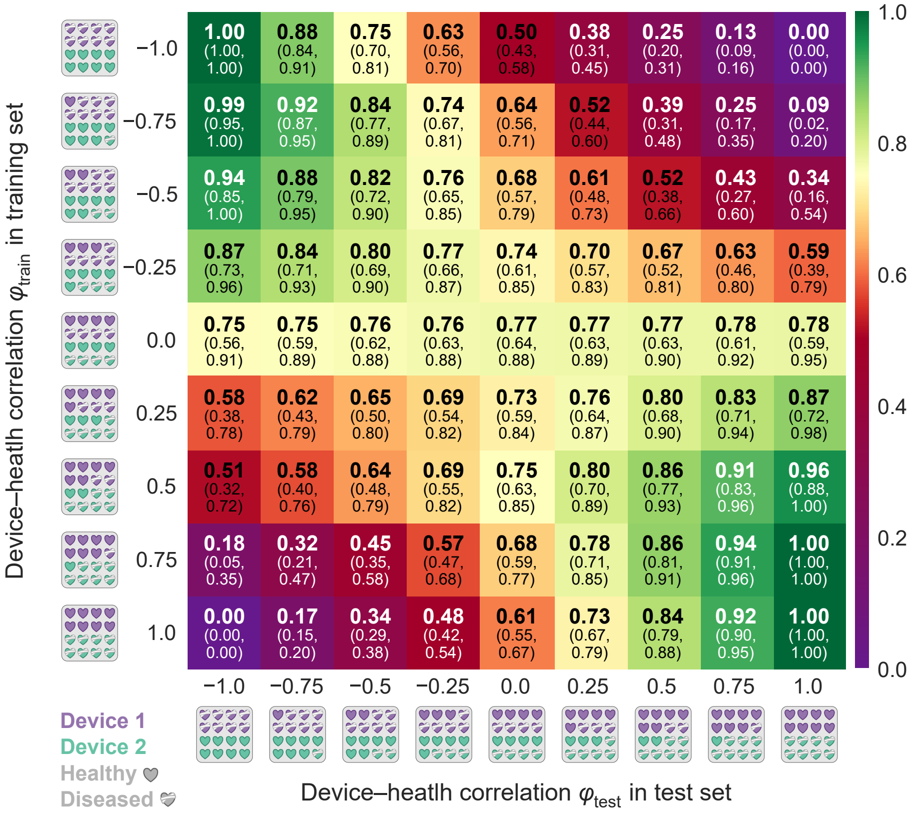

Figure S3. Increasing device–health correlation $|\varphi_{train}|$ reduces disease classifier robustness as measured by area under the receiver operating characteristic curve (AUROC). AUROC values are assessed for EfficientNetV2 disease classification models trained on diverse biased datasets with φ_{train} and computed for test sets with different φ_{test}. Each row in the table corresponds to models trained on a dataset featuring a specific device–health correlation φ_{train} during the training phase. A model exhibiting inconsistent performance across test sets with different φ_{test} indicates bias. This is evident in models trained with $\varphi_{train} \neq 0.0$ and especially for models trained with high $|\varphi_{train}|$. The bold values reflect the mean AUROC across the test set samples, with the values in brackets below representing the 95 % confidence intervals.

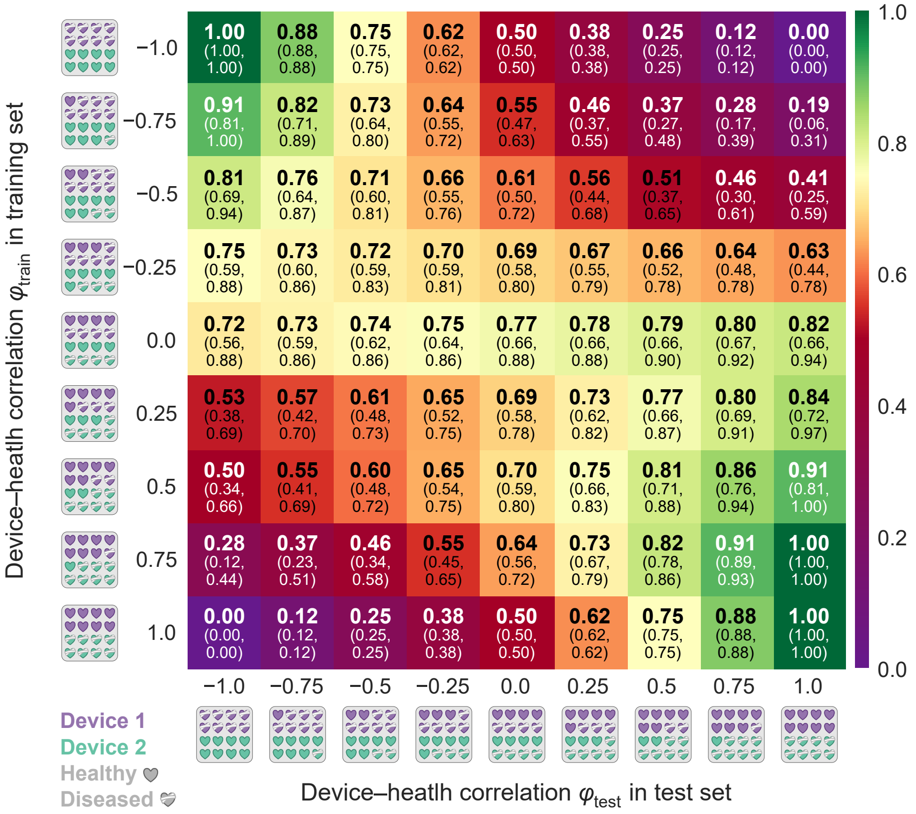

Figure S4. Increasing device–health correlation $|\varphi_{train}|$ reduces disease classifier robustness as measured by balanced accuracy (BA). BA values are assessed for EfficientNetV2 disease classification models trained on diverse biased datasets with φ_{train} and computed for test sets with different φ_{test}. Each row in the table corresponds to models trained on a dataset featuring a specific device–health correlation φ_{train} during the training phase. A model exhibiting inconsistent performance across test sets with different φ_{test} indicates bias. This is especially evident for models trained with high $|\varphi_{train}|$. The bold values reflect the mean BA across the test set samples, with the values in brackets below representing the 95 % confidence intervals.

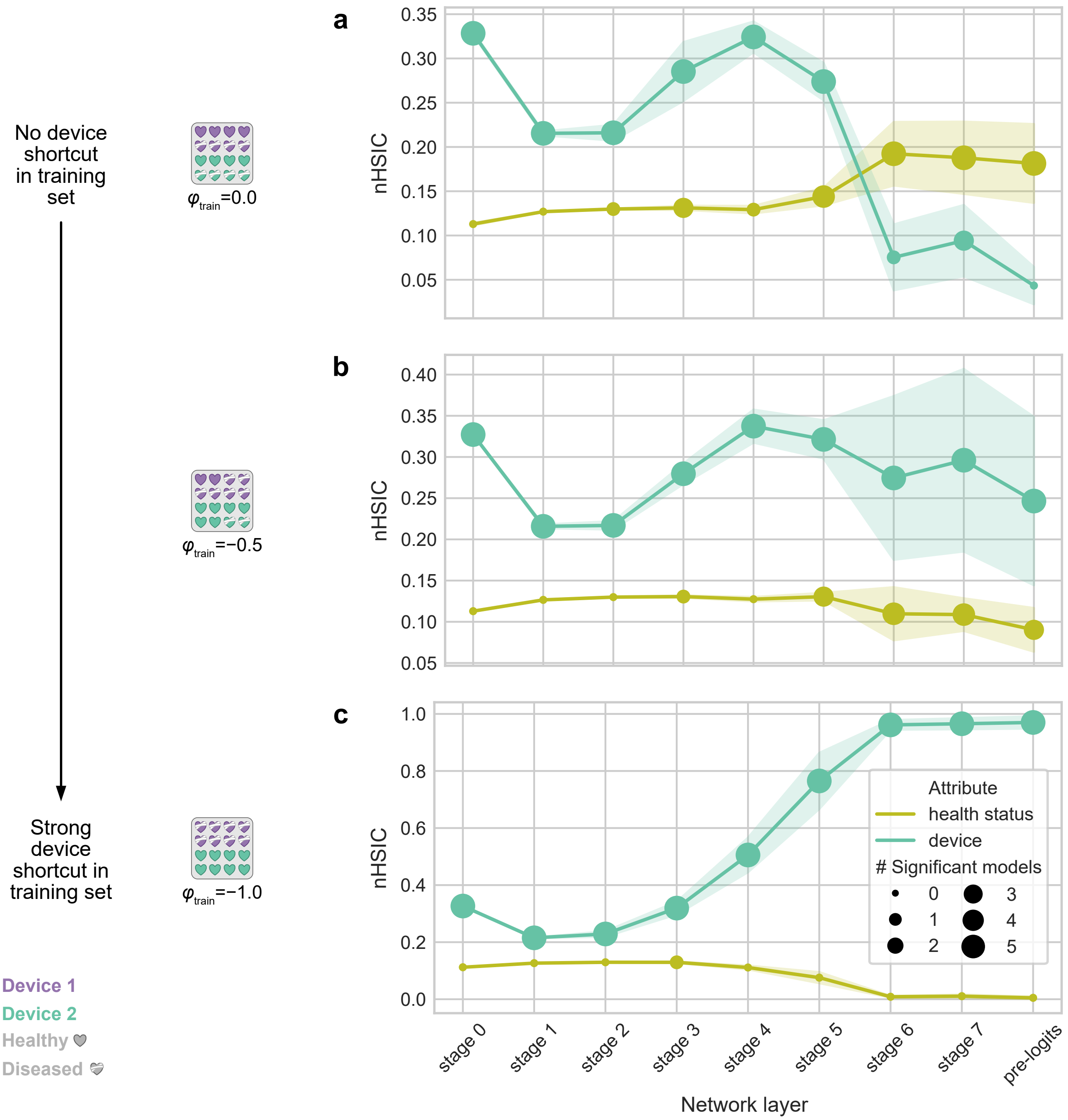

Figure S5. Device shortcuts in the training data trigger models to encode device information over health status across network depth. Feature encodings of the test set samples were extracted from nine network layers for all ensemble members of the PAD classifiers trained with $\varphi_{\text{train}} = 0.0$, -0.5, and -1.0. For each layer, the normalized Hilbert-Schmidt Independence Criterion (nHSIC) was calculated separately with respect to device identity (nHSIC_D) and health status (nHSIC_Y). **a** For $\varphi_{\text{train}} = 0.0$, nHSIC_D decreased notably in deeper layers, while nHSIC_Y increased and became significant across all ensemble members. **c** For $\varphi_{\text{train}} = -1.0$, nHSIC_D increased toward the final layers, whereas nHSIC_Y became zero. **b** For $\varphi_{\text{train}} = -0.5$, an intermediate pattern between those observed for $\varphi_{\text{train}} = 0.0$ and $\varphi_{\text{train}} = -1.0$ was observed, with nHSIC_D remaining higher than nHSIC_Y across network depth. Each data point shows the mean nHSIC across ensemble members (n=5) and the errorband the standard deviation across the members. Marker size denotes how many of the five ensemble members showed significant dependence. "Pre-logits" denotes the final representation before the classification head, and network stages 0-7 follow the EfficientNetV2-S architecture described in Table 4 of the original paper[2].

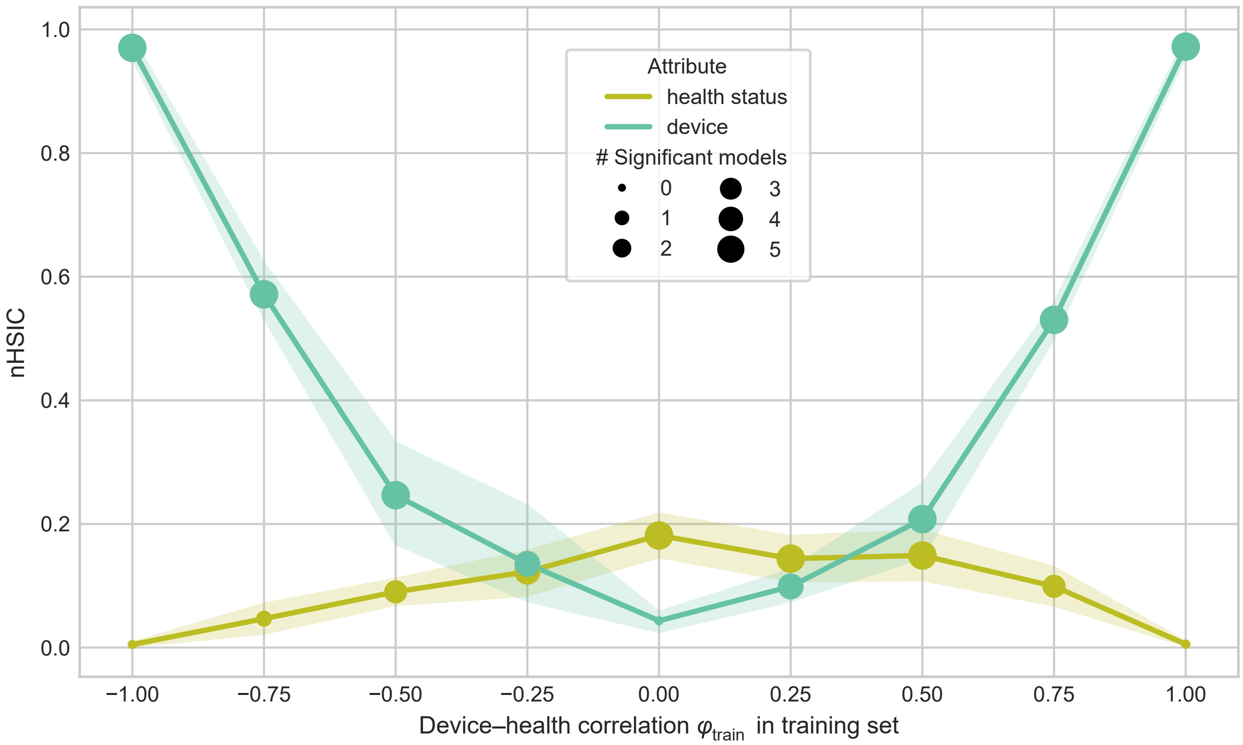

Figure S6. Final-layer representations increasingly encode device instance over health status with stronger device shortcuts during training. Normalized Hilbert–Schmidt Independence Criterion (nHSIC) values were computed separately for the device instance (nHSIC_D) and health status attributes (nHSIC_Y) on the pre-logit representations of the test set across disease classifier ensemble members trained at varying φ_{train}. Each data point shows the mean nHSIC across ensemble members ($n = 5$). Error bands show standard deviation across ensembles; marker size indicates the number of members (out of five) with significant dependence (permutation test, 1,000 repetitions). nHSIC_D rises near-quadratically with shortcut strength $|\varphi_{\text{train}}|$ and dominates nHSIC_Y for $|\varphi_{\text{train}}| > 0.25$, while nHSIC_Y increases as $|\varphi_{\text{train}}|$ decreases.

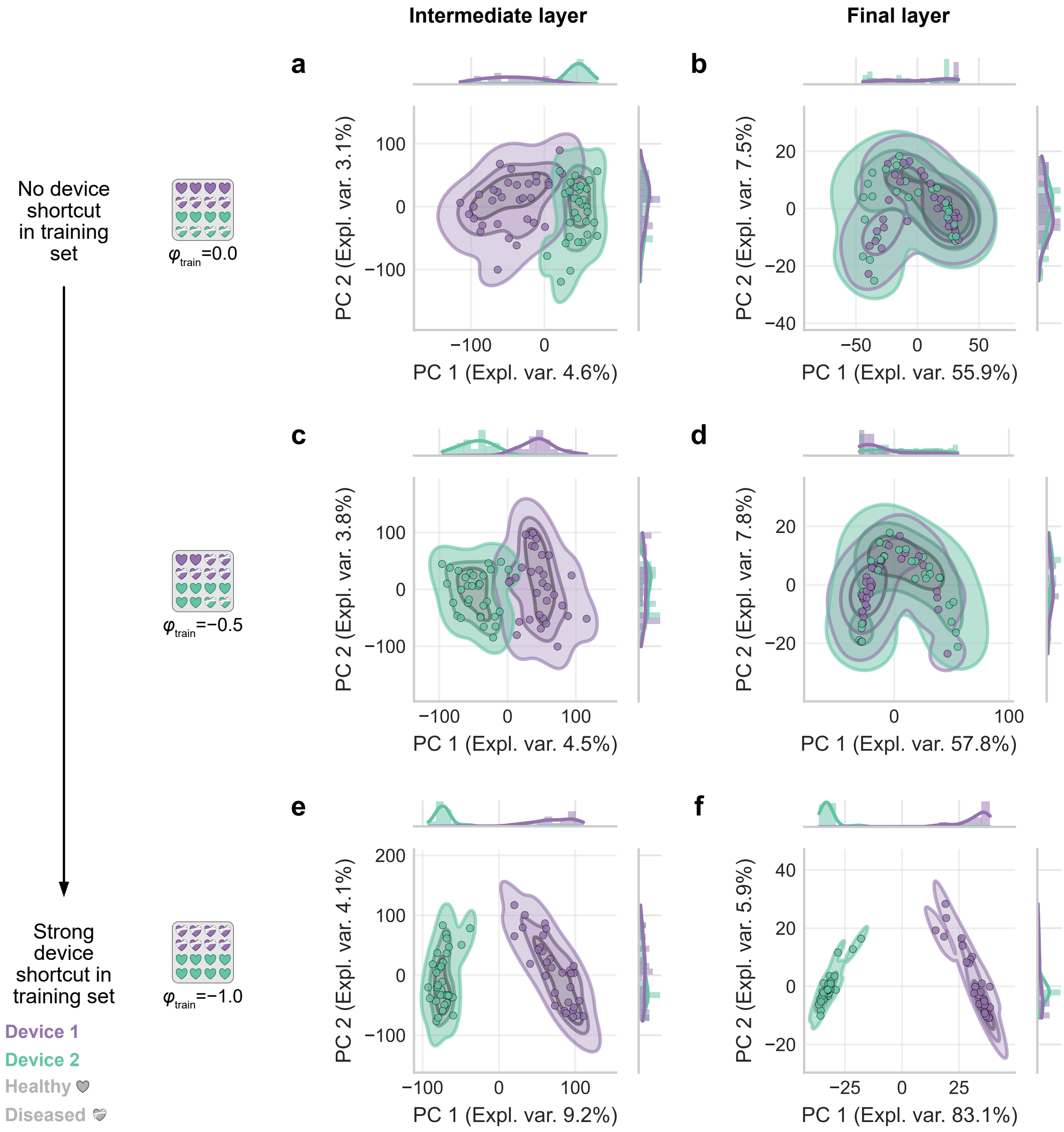

Figure S7. Device encodings persist across network depth when device shortcuts during training are strong. Shown are the projections on the first two PCs of z-score-normalized feature encodings from the test set samples, extracted from one representative ensemble member of disease classifiers trained with different φ_{train} colored by device instance. The left column (**a**,**c**,**e**) shows an intermediate representation after EfficientNetV2-S stage 5, and the right column (**b**,**d**,**f**) shows the final pre-logit representation. Intermediate-layer encodings cluster by device across all settings φ_{train} with larger separation for higher $|\varphi_{train}|$. In the final layer, separation by device was reduced when $\varphi_{train} = 0.0$, whereas high $|\varphi_{train}|$ led to stronger separation. The representative model was chosen as the ensemble member with median validation area under the receiver operating characteristic curve performance for the corresponding training setting AUROC($\varphi = \varphi_{train}$).

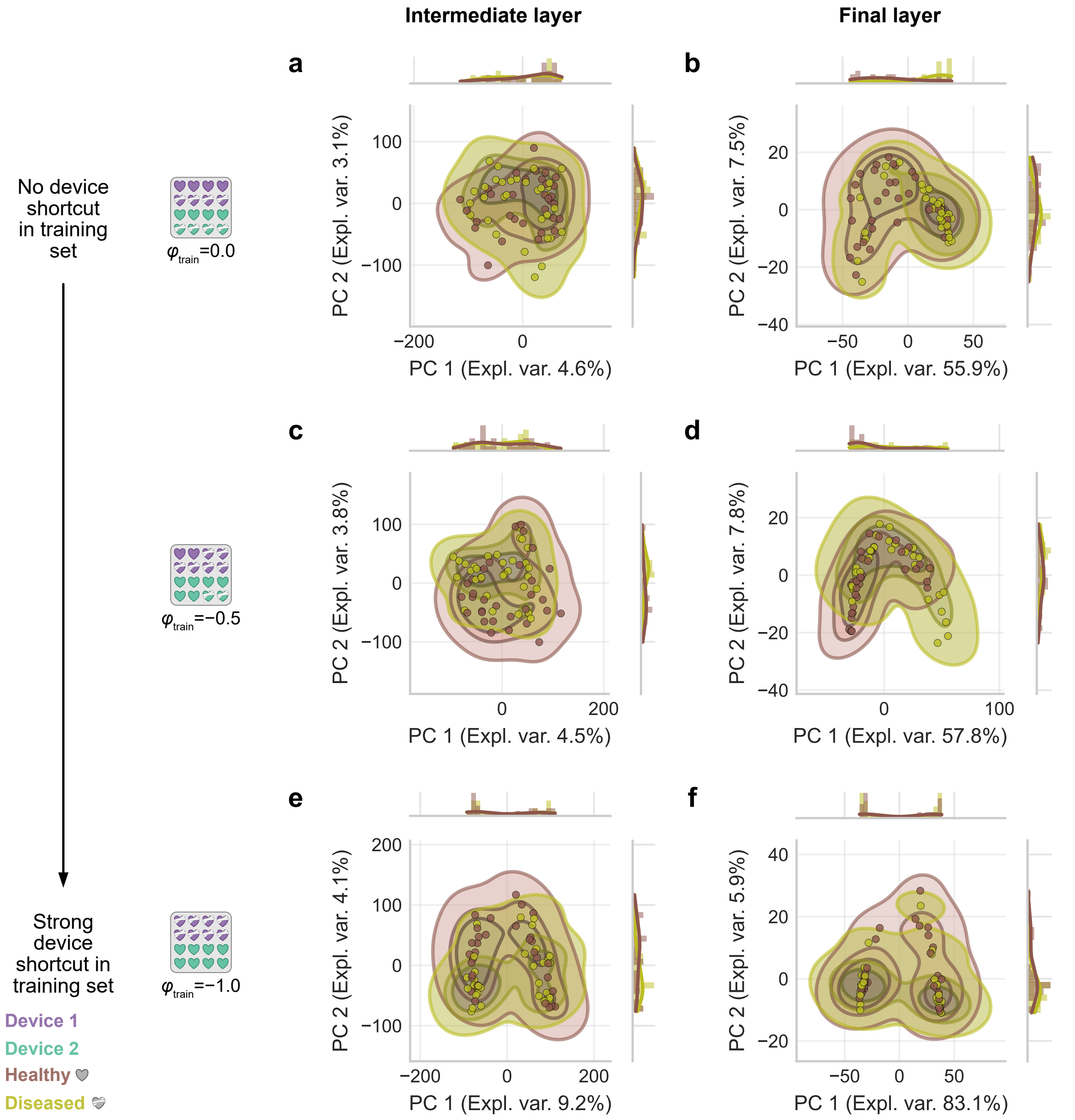

Figure S8. **Slight health status clustering emerges in final layers only under weak device shortcuts during training.** Shown are the projections on the first two PCs of z-score-normalized feature encodings from the test set samples, extracted from one representative ensemble member of disease classifiers trained with different φ_{train} colored by health status. The left column (**a**,**c**,**e**) shows an intermediate representation after EfficientNetV2-S stage 5, and the right column (**b**,**d**,**f**) shows the final pre-logit representation. Intermediate layers show no clear health status clustering across settings. Final-layer clustering by health status is absent under strong shortcuts ($|\varphi_{\text{train}}| > 0.5$) but emerges slightly for lower φ_{train}. The representative model was chosen as the ensemble member with median validation area under the receiver operating characteristic curve performance for the corresponding training setting AUROC($\varphi = \varphi_{\text{train}}$).

3.3 Shortcut learning phenomenon generalizes to transformer-based models

Parameter	Value
Dropout rate (classification head)	$\mathbb{P}_{\text{dropout}} = 0.15$
Batch size	$B = 16$
Learning rate	$\eta = 0.00001$
Gaussian noise augmentation	$\varepsilon = 0.001$

Table S7. Optimized hyperparameters for SwinV2-based disease diagnosis models. Unless stated otherwise, hyperparameter tuning and definitions and training protocol were identical to those in Table S6; only the optimized values listed here differed for the SwinV2 architecture.

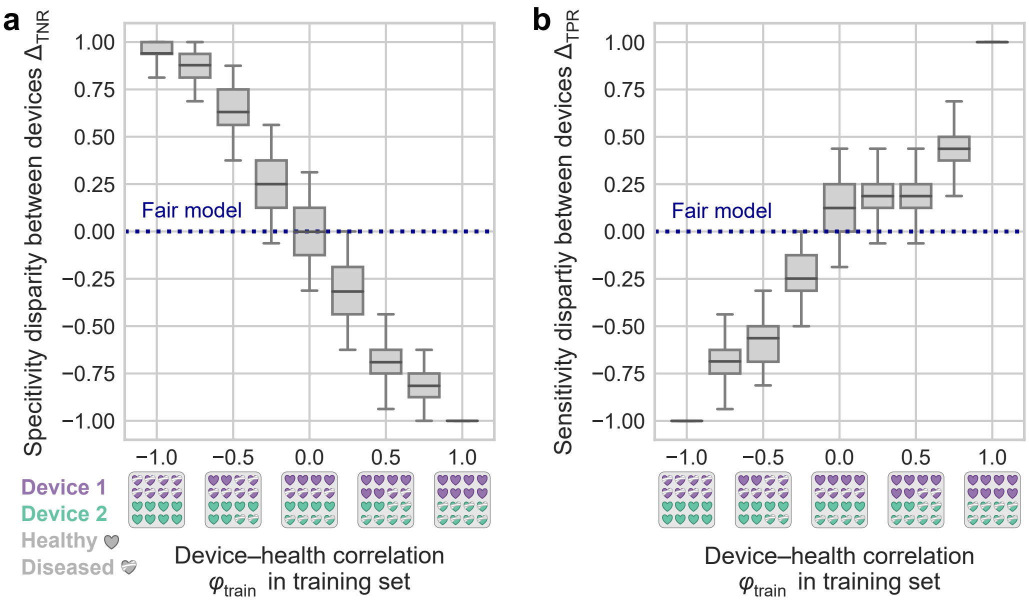

Figure S9. Stronger device shortcuts in the training data produce increasingly biased SwinV2 disease classifiers. Swin V2 model ensembles were trained to predict the health status for different levels of health status-device correlation in the training data. **a** Specificity disparity (Δ_{TNR}) and **b** sensitivity disparity (Δ_{TPR}) quantify fairness across device subgroups. A value of $\Delta = 0$ (blue dotted line) indicates perfect fairness, meaning equal performance for both devices. Model ensembles trained without device shortcuts showed no notable deviation from $\Delta = 0$. As $|\varphi_{\text{train}}|$ increased, the absolute disparity in both metrics grew, reaching a disparity $|\Delta| = 1$ under maximal correlation. Whiskers represent 95 % confidence intervals.

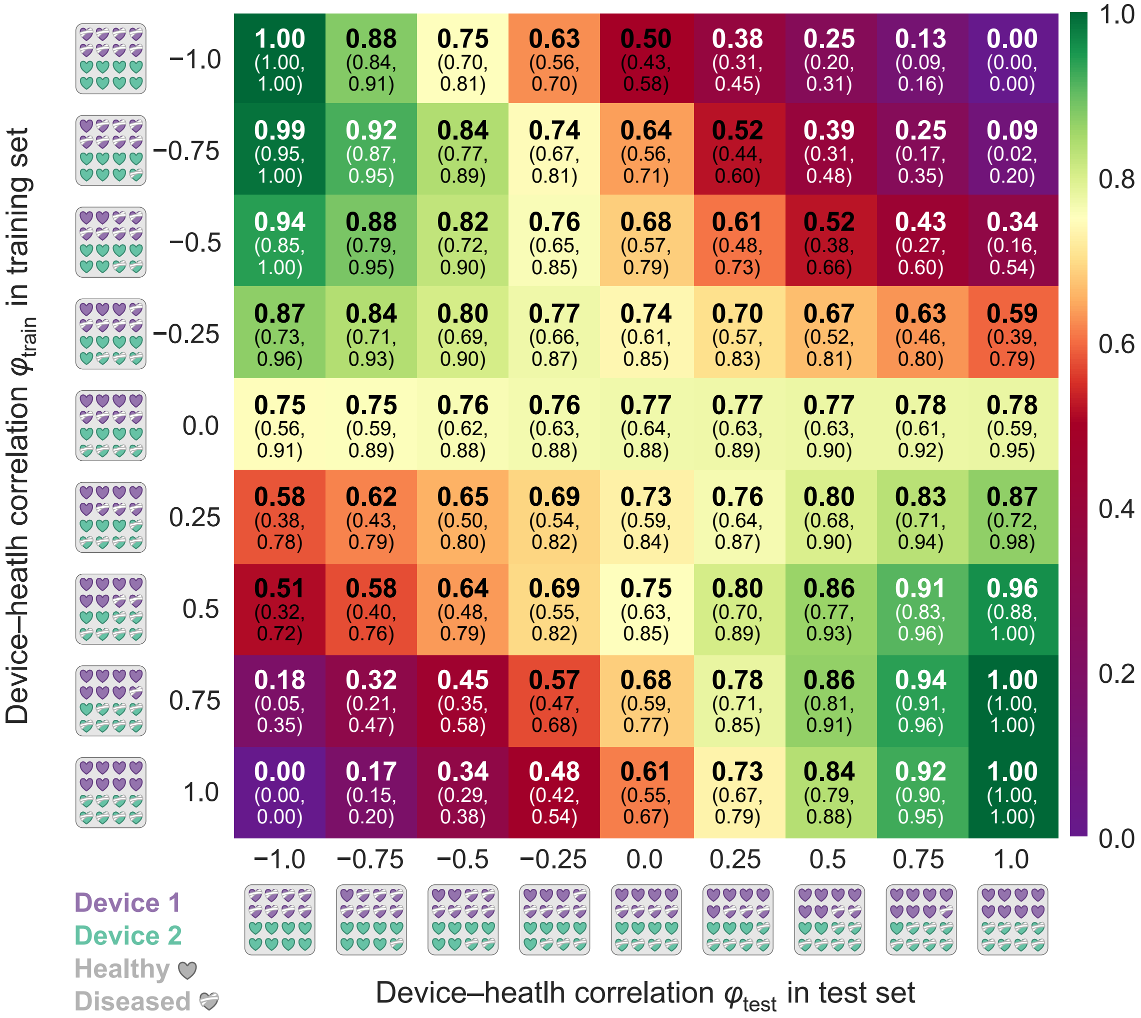

Figure S10. Increasing device–health correlation $|\varphi_{train}|$ reduces SwinV2 disease classifier robustness as measured by area under the receiver operating characteristic curve (AUROC). AUROC values are assessed for SwinV2 disease classification models trained on diverse biased datasets with φ_{train} and computed for test sets with different φ_{test}. Each row in the table corresponds to models trained on a dataset featuring a specific device–health correlation φ_{train} during the training phase. A model exhibiting inconsistent performance across test sets with different φ_{test} indicates bias. This is evident in models trained with $\varphi_{train} \neq 0.0$ and especially for models trained with high $|\varphi_{train}|$. The bold values reflect the mean AUROC across the test set samples, with the values in brackets below representing the 95 % confidence intervals.

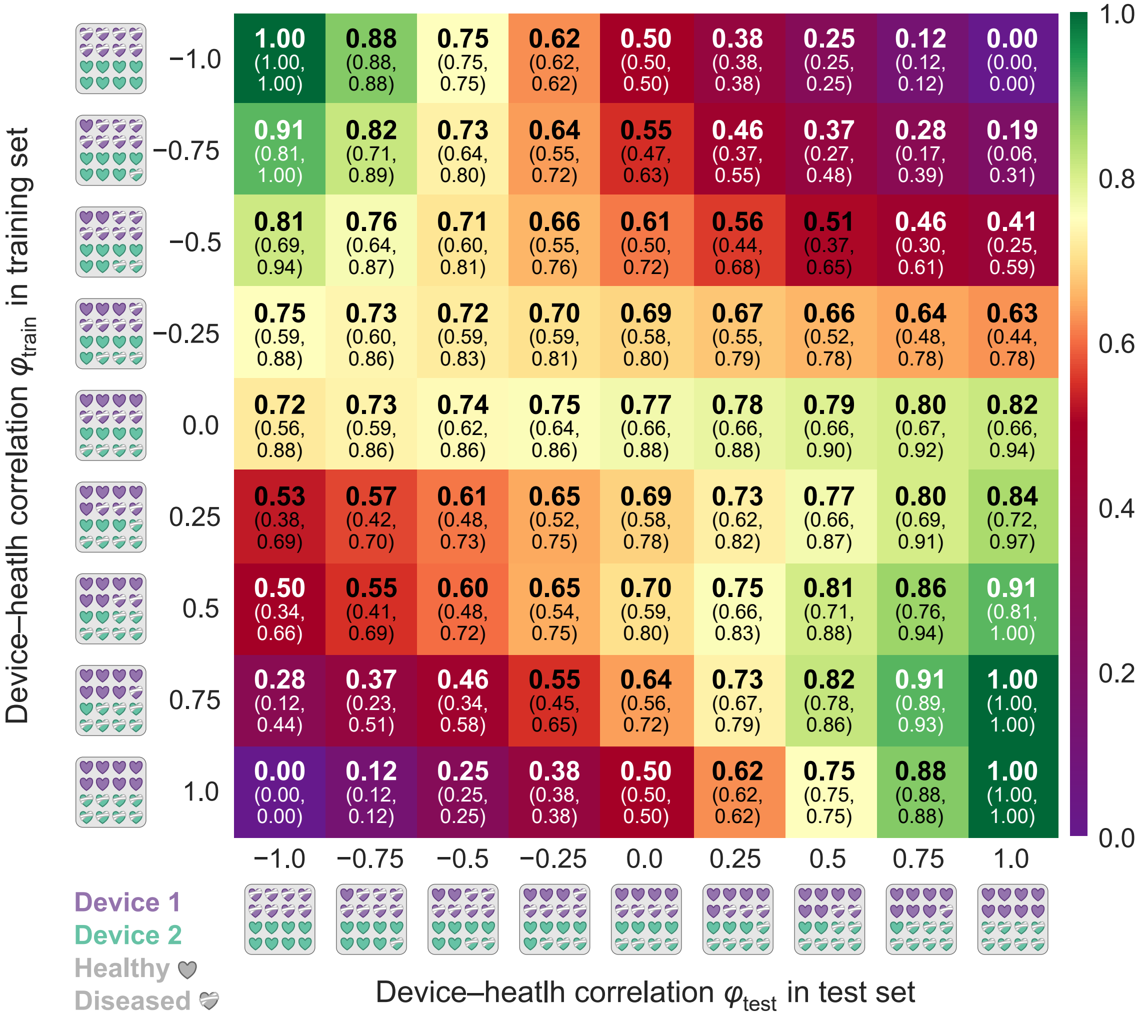

Figure S11. **Increasing device–health correlation $|\varphi_{train}|$ reduces SwinV2 disease classifier robustness as measured by balanced accuracy (BA).** BA values are assessed for SwinV2 disease classification models trained on diverse biased datasets with φ_{train} and computed for test sets with different φ_{test}. Each row in the table corresponds to models trained on a dataset featuring a specific device–health correlation φ_{train} during the training phase. A model exhibiting inconsistent performance across test sets with different φ_{test} indicates bias. This is especially evident for models trained with high $|\varphi_{train}|$. The bold values reflect the mean BA across the test set samples, with the values in brackets below representing the 95 % confidence intervals.

4 Proofs

Lemma 1: Relationship between φ and the device–health strata occurrences

The relationship between φ and the device–health strata occurrences within the test set are given by

$$\mathbb{P}(D{=}1 \mid Y^*{=}\text{healthy}) = \frac{1-\varphi}{2}, \tag{1}$$
$$\mathbb{P}(D{=}1 \mid Y^*{=}\text{diseased}) = \frac{1+\varphi}{2}, \tag{2}$$
$$\mathbb{P}(D{=}2 \mid Y^*{=}\text{healthy}) = \frac{1+\varphi}{2}, \tag{3}$$
$$\mathbb{P}(D{=}2 \mid Y^*{=}\text{diseased}) = \frac{1-\varphi}{2}, \tag{4}$$

if the condition of balanced device and health marginals holds, i.e.

$$N_1 = N_2 = N_{\text{healthy}} = N_{\text{diseased}} = \frac{N}{2}, \text{ and thus} \tag{5}$$
$$\mathbb{P}(D{=}1) = \mathbb{P}(D{=}2) = \mathbb{P}(Y^*{=}\text{healthy}) = \mathbb{P}(Y^*{=}\text{diseased}) = 0.5. \tag{6}$$

Here, N denotes the total number of samples in the test set.

Proof. By definition we have the following relationships

$$N := N_{1,\text{healthy}} + N_{2,\text{healthy}} + N_{1,\text{diseased}} + N_{2,\text{diseased}}, \tag{7}$$
$$N_1 := N_{1,\text{healthy}} + N_{1,\text{diseased}}, \tag{8}$$
$$N_2 := N_{2,\text{healthy}} + N_{2,\text{diseased}}, \tag{9}$$
$$N_{\text{healthy}} := N_{1,\text{healthy}} + N_{2,\text{healthy}}, \tag{10}$$
$$N_{\text{diseased}} := N_{1,\text{diseased}} + N_{2,\text{diseased}}. \tag{11}$$

Thus, applying (5), we equate the right-hand sides of (9) and (11) to obtain

$$N_{2,\text{healthy}} = N_{1,\text{diseased}}. \tag{12}$$

Similarly, equating the right-hand sides of (8) and (11) yields

$$N_{1,\text{healthy}} = N_{2,\text{diseased}}. \tag{13}$$

We can rewrite φ

$$\varphi \overset{\text{def}}{=} \frac{N_{1,\text{diseased}} N_{2,\text{healthy}} - N_{2,\text{diseased}} N_{1,\text{healthy}}}{\sqrt{N_1 N_2 N_{\text{diseased}} N_{\text{healthy}}}} \tag{14}$$
$$\overset{(5)}{=} \frac{N_{1,\text{diseased}} N_{2,\text{healthy}} - N_{2,\text{diseased}} N_{1,\text{healthy}}}{(N/2)^2} \tag{15}$$
$$\overset{(12)\wedge(13)}{=} \frac{N_{1,\text{diseased}}^2 - N_{2,\text{diseased}}^2}{(N/2)^2} \tag{16}$$
$$= \frac{N_{1,\text{diseased}}^2 + N_{1,\text{diseased}} N_{2,\text{diseased}} - N_{2,\text{diseased}} N_{1,\text{diseased}} - N_{2,\text{diseased}}^2}{(N/2)^2} \tag{17}$$
$$= \frac{N_{1,\text{diseased}} \left(N_{1,\text{diseased}} + N_{2,\text{diseased}}\right) - N_{2,\text{diseased}} \left(N_{1,\text{diseased}} + N_{2,\text{diseased}}\right)}{(N/2)^2} \tag{18}$$
$$\overset{(11)\wedge(5)}{=} \frac{N_{1,\text{diseased}}}{N_{\text{diseased}}} - \frac{N_{2,\text{diseased}}}{N_{\text{diseased}}}. \tag{19}$$

These fractions can be interpreted as conditional probabilities via the counting definition of probabilities and thus

$$\varphi \overset{(19)}{=} \mathbb{P}(D{=}1 \mid Y^*{=}\text{diseased}) - \mathbb{P}(D{=}2 \mid Y^*{=}\text{diseased}). \tag{20}$$

Due to the law of total probability, we have for the binary case $d \in \{1,2\}, y^* \in \{\text{healthy}, \text{diseased}\}$

$$\forall y^* : \mathbb{P}(D{=}1 \mid Y^*{=}y^*) = 1 - \mathbb{P}(D{=}2 \mid Y^*{=}y^*) \tag{21}$$
$$\forall d : \mathbb{P}(Y^*{=}\text{healthy} \mid D) = 1 - \mathbb{P}(Y^*{=}\text{diseased} \mid D) \tag{22}$$

and thus one can rewrite (20)

$$\varphi \overset{(21)}{=} 2\mathbb{P}(D{=}1 \mid Y^*{=}\text{diseased}) - 1, \text{ and} \tag{23}$$
$$\varphi \overset{(21)}{=} 1 - 2\mathbb{P}(D{=}2 \mid Y^*{=}\text{diseased}). \tag{24}$$

Given (6) one can use Bayes' Theorem to derive

$$\forall d, y^* : \mathbb{P}(D{=}d \mid Y^*{=}y^*) = \frac{\mathbb{P}(Y^*{=}y^* \mid D{=}d)\,\mathbb{P}(D{=}d)}{\mathbb{P}(Y^*{=}y^*)} \overset{(6)}{=} \mathbb{P}(Y^*{=}y^* \mid D{=}d), \tag{25}$$

and thus

$$\varphi \overset{(25)}{=} 2\mathbb{P}(Y^*{=}\text{diseased} \mid D{=}1) - 1 \overset{(22)}{=} 1 - 2\mathbb{P}(Y^*{=}\text{healthy} \mid D{=}1) \overset{(25)}{=} 1 - 2\mathbb{P}(D{=}1 \mid Y^*{=}\text{healthy}), \tag{26}$$
$$\varphi \overset{(25)}{=} 1 - 2\mathbb{P}(Y^*{=}\text{diseased} \mid D{=}2) \overset{(22)}{=} 2\mathbb{P}(Y^*{=}\text{healthy} \mid D{=}2) - 1 \overset{(25)}{=} 2\mathbb{P}(D{=}2 \mid Y^*{=}\text{healthy}) - 1. \tag{27}$$

Rewriting eq. (23), eq. (24) eq. (26) and eq. (27) gives what we wanted to show

$$\mathbb{P}(D{=}1 \mid Y^*{=}\text{healthy}) = \frac{1-\varphi}{2}, \tag{28}$$
$$\mathbb{P}(D{=}1 \mid Y^*{=}\text{diseased}) = \frac{1+\varphi}{2}, \tag{29}$$
$$\mathbb{P}(D{=}2 \mid Y^*{=}\text{healthy}) = \frac{1+\varphi}{2}, \tag{30}$$
$$\mathbb{P}(D{=}2 \mid Y^*{=}\text{diseased}) = \frac{1-\varphi}{2}. \tag{31}$$

□

Lemma 2: φ-dependency of the sensitivity and specificity

The φ-dependency of the sensitivity and specificity is given by

$$\text{TNR}(\varphi) = \frac{1-\varphi}{2}\text{TNR}\big|_1 + \frac{1+\varphi}{2}\text{TNR}\big|_2, \text{ and} \tag{32}$$
$$\text{TPR}(\varphi) = \frac{1+\varphi}{2}\text{TPR}\big|_1 + \frac{1-\varphi}{2}\text{TPR}\big|_2, \tag{33}$$

assuming the balanced device and health marginals condition from Lemma 1.

Proof. Let $\widehat{Y}$ be the predicted health status. One can derive

$$\mathbb{P}(\widehat{Y} \mid Y^*) = \frac{\mathbb{P}(\widehat{Y}, Y^*)}{\mathbb{P}(Y^*)} = \sum_d \frac{\mathbb{P}(\widehat{Y}, Y^*, D{=}d)}{\mathbb{P}(Y^*)} = \sum_d \frac{\mathbb{P}(\widehat{Y} \mid Y^*, D{=}d)\,\mathbb{P}(D{=}d \mid Y^*)\,\mathbb{P}(Y^*)}{\mathbb{P}(Y^*)} \tag{34}$$
$$= \sum_d \mathbb{P}(\widehat{Y} \mid Y^*, D{=}d)\,\mathbb{P}(D{=}d \mid Y^*). \tag{35}$$

In the main paper sensitivity and specificity were defined conditioned for each subgroup as

$$\text{TPR}\big|_d := \mathbb{P}(\widehat{Y}{=}\text{diseased} \mid Y^*{=}\text{diseased}, D{=}d), \quad \text{TNR}\big|_d := \mathbb{P}(\widehat{Y}{=}\text{healthy} \mid Y^*{=}\text{healthy}, D{=}d). \tag{36}$$

The eq. (35) has the following implications for the sensitivity and specificity

$$\begin{aligned}
\mathrm{TPR} &= \mathbb{P}(Y^*{=}\text{diseased} \mid Y^*{=}\text{diseased}) && (37)\\
&\overset{(35)}{=} \mathbb{P}(Y^*{=}\text{diseased} \mid Y^*{=}\text{diseased}, D{=}1)\,\mathbb{P}(D{=}1 \mid Y^*{=}\text{diseased})\\
&\quad + \mathbb{P}(Y^*{=}\text{diseased} \mid Y^*{=}\text{diseased}, D{=}2)\,\mathbb{P}(D{=}2 \mid Y^*{=}\text{diseased}) && (38)\\
&\overset{\text{def}}{=} \mathrm{TPR}\big|_1\,\mathbb{P}(D{=}1 \mid Y^*{=}\text{diseased}) + \mathrm{TPR}\big|_2\,\mathbb{P}(D{=}2 \mid Y^*{=}\text{diseased}) && (39)\\
&\overset{(2)\wedge(4)}{=} \frac{1+\varphi}{2}\mathrm{TPR}\big|_1 + \frac{1-\varphi}{2}\mathrm{TPR}\big|_2, && (40)
\end{aligned}$$

and analogously

$$\begin{aligned}
\mathrm{TNR} &= \mathbb{P}(\widehat{Y}{=}\text{healthy} \mid Y^*{=}\text{healthy}) && (41)\\
&\overset{(35)}{=} \mathbb{P}(\widehat{Y}{=}\text{healthy} \mid Y^*{=}\text{healthy}, D{=}1)\,\mathbb{P}(D{=}1 \mid Y^*{=}\text{healthy})\\
&\quad + \mathbb{P}(\widehat{Y}{=}\text{healthy} \mid Y^*{=}\text{healthy}, D{=}2)\,\mathbb{P}(D{=}2 \mid Y^*{=}\text{healthy}) && (42)\\
&\overset{\text{def}}{=} \mathrm{TNR}\big|_1\,\mathbb{P}(D{=}1 \mid Y^*{=}\text{healthy}) + \mathrm{TNR}\big|_2\,\mathbb{P}(D{=}2 \mid Y^*{=}\text{healthy}) && (43)\\
&\overset{(1)\wedge(3)}{=} \frac{1-\varphi}{2}\mathrm{TNR}\big|_1 + \frac{1+\varphi}{2}\mathrm{TNR}\big|_2 && (44)
\end{aligned}$$

□

Lemma 3: φ-Dependency of AUROC

The expression for the φ-dependent area under receiver operating characteristic curve (AUROC) is given by

$$\begin{aligned}
\mathrm{AUROC}(\varphi) = \frac{1}{8}\sum_{j=0}^{|C_{\text{thr}}|-1} \Big\{ &\big[(1+\varphi)(\mathrm{TPR}_j\big|_1 + \mathrm{TPR}_{j+1}\big|_1) + (1-\varphi)(\mathrm{TPR}_j\big|_2 + \mathrm{TPR}_{j+1}\big|_2)\big]\\
&\cdot \big[(1-\varphi)(\mathrm{TNR}_j\big|_1 - \mathrm{TNR}_{j+1}\big|_1) + (1+\varphi)(\mathrm{TNR}_j\big|_2 - \mathrm{TNR}_{j+1}\big|_2)\big]\Big\},
\end{aligned} \qquad (45)$$

assuming the balanced device and health marginals condition from Lemma 1.

Proof. Let $\hat{\mathbb{P}}(x)$ be the calibrated predicted disease probability of the trained model for input x, and define the hard prediction at threshold c as

$$\hat{Y}_c(x) := \begin{cases} 1, & \hat{\mathbb{P}}(x) \geq c,\\ 0, & \hat{\mathbb{P}}(x) < c. \end{cases}$$

For the subgroup with device $d \in \{1,2\}$, define the confusion-matrix counts at threshold c^j_{thr} as

$$\begin{aligned}
\mathrm{TP}_j\big|_d &:= \#\{i \mid D_i = d,\ Y_i = 1,\ \hat{\mathbb{P}}(x_i) \geq c^j_{\text{thr}}\},\\
\mathrm{FN}_j\big|_d &:= \#\{i \mid D_i = d,\ Y_i = 1,\ \hat{\mathbb{P}}(x_i) < c^j_{\text{thr}}\},\\
\mathrm{TN}_j\big|_d &:= \#\{i \mid D_i = d,\ Y_i = 0,\ \hat{\mathbb{P}}(x_i) < c^j_{\text{thr}}\},\\
\mathrm{FP}_j\big|_d &:= \#\{i \mid D_i = d,\ Y_i = 0,\ \hat{\mathbb{P}}(x_i) \geq c^j_{\text{thr}}\}.
\end{aligned}$$

Then the subgroup-specific true positive rate (TPR) and true negative rate (TNR) at threshold c^j_{thr} are

$$\mathrm{TPR}_j\big|_d := \frac{\mathrm{TP}_j\big|_d}{\mathrm{TP}_j\big|_d + \mathrm{FN}_j\big|_d} = \Pr\big(\hat{Y}_{c^j_{\text{thr}}} = 1 \,\big|\, Y = 1,\ D = d\big),$$

$$\mathrm{TNR}_j\big|_d := \frac{\mathrm{TN}_j\big|_d}{\mathrm{TN}_j\big|_d + \mathrm{FP}_j\big|_d} = \Pr\big(\hat{Y}_{c^j_{\text{thr}}} = 0 \,\big|\, Y = 0,\ D = d\big).$$

One can derive

$$\mathrm{AUROC}(\varphi) = \sum_{j=0}^{|C_{\mathrm{thr}}|-1} \frac{\mathrm{TPR}_j + \mathrm{TPR}_{j+1}}{2} \cdot (\mathrm{FPR}_{j+1} - \mathrm{FPR}_j) \tag{46}$$

$$\overset{(40)}{=} \frac{1}{2} \sum_{j=0}^{|C_{\mathrm{thr}}|-1} \left[\left\{ \frac{1+\varphi}{2} \mathrm{TPR}_j\big|_1 + \frac{1-\varphi}{2} \mathrm{TPR}_j\big|_2 + \frac{1+\varphi}{2} \mathrm{TPR}_{j+1}\big|_1 + \frac{1-\varphi}{2} \mathrm{TPR}_{j+1}\big|_2 \right\} \left\{ \mathrm{FPR}_{j+1} - \mathrm{FPR}_j \right\} \right] \tag{47}$$

$$\overset{\mathrm{def}}{=} \frac{1}{2} \sum_{j=0}^{|C_{\mathrm{thr}}|-1} \left[\left\{ \frac{1+\varphi}{2} \mathrm{TPR}_j\big|_1 + \frac{1-\varphi}{2} \mathrm{TPR}_j\big|_2 + \frac{1+\varphi}{2} \mathrm{TPR}_{j+1}\big|_1 + \frac{1-\varphi}{2} \mathrm{TPR}_{j+1}\big|_2 \right\} \left\{ (1 - \mathrm{TNR}_{j+1}) - (1 - \mathrm{TNR}_j) \right\} \right] \tag{48}$$

$$= \frac{1}{2} \sum_{j=0}^{|C_{\mathrm{thr}}|-1} \left[\left\{ \frac{1+\varphi}{2} \mathrm{TPR}_j\big|_1 + \frac{1-\varphi}{2} \mathrm{TPR}_j\big|_2 + \frac{1+\varphi}{2} \mathrm{TPR}_{j+1}\big|_1 + \frac{1-\varphi}{2} \mathrm{TPR}_{j+1}\big|_2 \right\} \left\{ \mathrm{TNR}_j - \mathrm{TNR}_{j+1} \right\} \right] \tag{49}$$

$$\overset{(44)}{=} \frac{1}{2} \sum_{j=0}^{|C_{\mathrm{thr}}|-1} \left[\left\{ \frac{1+\varphi}{2} \mathrm{TPR}_j\big|_1 + \frac{1-\varphi}{2} \mathrm{TPR}_j\big|_2 + \frac{1+\varphi}{2} \mathrm{TPR}_{j+1}\big|_1 + \frac{1-\varphi}{2} \mathrm{TPR}_{j+1}\big|_2 \right\} \left\{ \frac{1-\varphi}{2} \mathrm{TNR}_j\big|_1 + \frac{1+\varphi}{2} \mathrm{TNR}_j\big|_2 - \frac{1-\varphi}{2} \mathrm{TNR}_{j+1}\big|_1 - \frac{1+\varphi}{2} \mathrm{TNR}_{j+1}\big|_2 \right\} \right] \tag{50}$$

$$= \frac{1}{8} \sum_{j=0}^{|C_{\mathrm{thr}}|-1} \left[\left\{ (1+\varphi) \left(\mathrm{TPR}_j\big|_1 + \mathrm{TPR}_{j+1}\big|_1 \right) + (1-\varphi) \left(\mathrm{TPR}_j\big|_2 + \mathrm{TPR}_{j+1}\big|_2 \right) \right\} \left\{ (1-\varphi) \left(\mathrm{TNR}_j\big|_1 - \mathrm{TNR}_{j+1}\big|_1 \right) + (1+\varphi) \left(\mathrm{TNR}_j\big|_2 - \mathrm{TNR}_{j+1}\big|_2 \right) \right\} \right]. \tag{51}$$

□

5 Pseudocode

Algorithm 1: Membrane signal-to-noise ratio computation

Let $I_{\lambda}^{(i)}(x,y)$ denote the reconstructed in aqua image of frame i at wavelength λ, with lateral coordinate x and depth coordinate y. This algorithm is applied for each device separately to get the SNR per device. The algorithm first identifies device-specific membrane pixels from the mean image at λ_{ref}, and subsequently computes the laser-energy-corrected membrane signal and corresponding signal-to-noise ratio for all frames and wavelengths of that device.

1) define membrane pixels to be used for signal definition;
compute the mean $\lambda_{\text{ref}} = 1210\,\text{nm}$ image;
$\bar{I}_{\lambda_{\text{ref}}}(x,y) \leftarrow \text{mean}_i\left(I_{\lambda_{\text{ref}}}^{(i)}(x,y)\right)$;
define C as the central 200 columns, corresponding to the inner 20 mm of the image width in order to minimize boundary effects and avoid membrane artifacts due to membrane degradations;
initialize membrane masks;
$M_{\text{upper}}(x,y) \leftarrow 0$;
$M_{\text{lower}}(x,y) \leftarrow 0$;
for *each column* $x \in C$ **do**
  extract the column profile;
  $p_x(y) \leftarrow \bar{I}_{\lambda_{\text{ref}}}(x,y)$;
  compute the adjacent-depth sum profile;
  $\tilde{p}_x(y) \leftarrow p_x(y) + p_x(y+1)$ for all valid y;
  normalize the profile by its maximum;
  $\hat{p}_x(y) \leftarrow \tilde{p}_x(y) / \max_y \tilde{p}_x(y)$;
  detect peaks with minimum height 0.6 and minimum distance 5 (using `scipy.signal.find_peaks`[3]);
  $P_x \leftarrow f_{\text{find\_peaks}}(\hat{p}_x(y), \text{height} = 0.6, \text{distance} = 5)$;
  assign the first two detected peaks to the membrane lines;
  $y_{\text{upper}}(x) \leftarrow P_x[0]$;
  $y_{\text{lower}}(x) \leftarrow P_x[1]$;
  add the corresponding two-pixel depth intervals to the masks;
  $M_{\text{upper}}(x, y_{\text{upper}}(x) : y_{\text{upper}}(x)+1) \leftarrow 1$;
  $M_{\text{lower}}(x, y_{\text{lower}}(x) : y_{\text{lower}}(x)+1) \leftarrow 1$;
end
2) compute signal-to-noise ratio for all samples and wavelengths;
for *each frame i and wavelength* λ_j **do**
  extract image $I_{\lambda_j}^{(i)}(x,y)$;
  compute membrane signal from the two membrane masks providing robustness against outliers and parasitic noise;
  $s_{\text{upper}}^{(i,j)} \leftarrow \text{median}\left(I_{\lambda_j}^{(i)}(x,y) \mid (x,y) \in M_{\text{upper}}\right)$;
  $s_{\text{lower}}^{(i,j)} \leftarrow \text{median}\left(I_{\lambda_j}^{(i)}(x,y) \mid (x,y) \in M_{\text{lower}}\right)$;
  $s_{\text{mem}}^{(i,j)} \leftarrow \left(s_{\text{upper}}^{(i,j)} + s_{\text{lower}}^{(i,j)}\right)/2$;
  correct membrane signal for laser pulse energy $E^{(i,j)}$;
  $s_{\text{mem},E\text{corr}}^{(i,j)} \leftarrow s_{\text{mem}}^{(i,j)} / E^{(i,j)}$;
  estimate image noise using a robust wavelet-based estimator (using `skimage.restoration.estimate_sigma` based on[4]);
  $\sigma_{\text{img}}^{(i,j)} \leftarrow f_{\text{estimate\_sigma}}\left(I_{\lambda_j}^{(i)}\right)$;
  compute signal-to-noise ratios;
  $\text{SNR}^{(i,j)} \leftarrow s_{\text{mem},E\text{corr}}^{(i,j)} / \sigma_{\text{img}}^{(i,j)}$;
end

Algorithm 2: Broken sensor interpolation

Let n_b be the broken sensor and n_l, n_r be nearest functioning sensors to the left and right, respectively. Let $S_n(t)$ be the raw time series signal of a sensor n at timestep $t \in \{1, \ldots, 2030\}$. Where each time step corresponds to 25 ns. The idea behind this algorithm is to estimate the time shift δt between the signals of n_l and n_r via cross-correlation and then to compute interpolated signal $\hat{S}_{n_b}(t)$ as the average of these neighboring signals while accounting for δt.

Center $S_{n_l}(t)$ and $S_{n_r}(t)$ around 0 via bandpassfiltering;
$\tilde{S}_{n_l}(t) \leftarrow f_{\text{Bandpassfilter}}(S_{n_l}(t))$;
$\tilde{S}_{n_r}(t) \leftarrow f_{\text{Bandpassfilter}}(S_{n_r}(t))$;
$R_{\text{best}} \leftarrow 0$;
for *shift* $\delta t \in \{0.0, 0.5, 1.0, 1.5, 2.0\}$ **do**
  shift signals using third-order spline (using `scipy.ndimage.shift`);
  $\tilde{S}_{n_l}(t) \leftarrow g_{\text{spline},3}(\tilde{S}_{n_l}, -\delta t)$;
  $\tilde{S}_{n_r}(t) \leftarrow g_{\text{spline},3}(\tilde{S}_{n_r}, \delta t)$;
  calculate cross-correlation R entry for this shift;
  $R \leftarrow \sum_{t>330}^{1730} \tilde{S}_{n_l}(t) \cdot \tilde{S}_{n_r}(t)$;
  if $R > R_{best}$ **then**
    $R_{\text{best}} \leftarrow R$;
    $\delta t_{\text{best}} \leftarrow \delta t$;
  end
end
calculate the best estimator for the broken sensor signal;
$\hat{S}_{n_b}(t) \leftarrow \left(g_{\text{spline},3}(S_{n_l}, -\delta t_{\text{best}}) + g_{\text{spline},3}(S_{n_r}, \delta t_{\text{best}})\right)/2$;

Algorithm 3: Early response sensor correction

Let n_{er} be an early response sensor. Let $S_{n_{er}}(t)$ be its raw time series at time step $t \in \{1, \ldots, 2030\}$, where each time step corresponds to 25 ns. Let $\hat{S}_{n_{er}}(t)$ be the reference signal obtained by interpolation from neighboring functioning sensors (see Algorithm 2). The idea behind this algorithm is to estimate the temporal offset δt between $S_{n_{er}}(t)$ and $\hat{S}_{n_{er}}(t)$ by cross-correlation and to shift the raw signal accordingly.

Estimate a reference signal from neighboring sensors via algorithm 2;
$\hat{S}_{n_{er}}(t) \leftarrow$ Algorithm 2;
Center $S_{n_{er}}(t)$ and $\hat{S}_{n_{er}}(t)$ around 0 via bandpassfiltering;
$\tilde{S}_{n_{er}}(t) \leftarrow f_{\text{Bandpassfilter}}(S_{n_{er}}(t))$;
$\tilde{\hat{S}}_{n_{er}}(t) \leftarrow f_{\text{Bandpassfilter}}(\hat{S}_{n_{er}}(t))$;
$R_{\text{best}} \leftarrow 0$;
for *shift* $\delta t \in \{-2.0, -1.5, -1.0, -0.5, 0.0, 0.5, 1.0, 1.5, 2.0\}$ **do**
  $\tilde{S}^{\delta}_{n_{er}}(t) \leftarrow g_{\text{spline},3}(\tilde{S}_{n_{er}}, \delta t)$;
  $R \leftarrow \sum_{t=331}^{1730} \tilde{S}^{\delta}_{n_{er}}(t) \cdot \tilde{\hat{S}}_{n_{er}}(t)$;
  if $R > R_{best}$ **then**
    $R_{\text{best}} \leftarrow R$;
    $\delta t_{\text{best}} \leftarrow \delta t$;
  end
end
correct the original early-response signal;
$S^{\text{corr}}_{n_{er}}(t) \leftarrow g_{\text{spline},3}(S_{n_{er}}, \delta t_{\text{best}})$;

References

1. Bender, C. J. *Hardware-Related Biases in Machine Learning Algorithms for Photoacoustic Image Analysis*. Master's thesis, Heidelberg University Faculty of Physics and Astronomy, Heidelberg, Germany (2023).
2. Tan, M. & Le, Q. EfficientNetV2: Smaller Models and Faster Training. In *Proceedings of the 38th International Conference on Machine Learning*, 10096–10106, DOI: 10.48550/arXiv.2104.00298 (2021).
3. Virtanen, P. *et al.* SciPy 1.0: Fundamental algorithms for scientific computing in Python. *Nat. Methods* **17**, 261–272, DOI: 10.1038/s41592-019-0686-2 (2020).
4. Donoho, D. L. & Johnstone, I. M. Ideal spatial adaptation by wavelet shrinkage. *Biometrika* **81**, 425–455, DOI: 10.1093/biomet/81.3.425 (1994).
